# Enhanced antibacterial and anticancer properties of Se-NPs decorated TiO_2_ nanotube film

**DOI:** 10.1371/journal.pone.0214066

**Published:** 2019-03-22

**Authors:** Ondrej Bilek, Zdenka Fohlerova, Jaromir Hubalek

**Affiliations:** 1 Central European Institute of Technology, Brno University of Technology, Brno, Czech Republic; 2 Department of Microelectronics, Brno University of Technology, Brno, Czech Republic; Northeastern University, UNITED STATES

## Abstract

Selenium nanoparticle modified surfaces attract increasing attention in the field of tissue engineering. Selenium exhibits strong anticancer, antibacterial and anti-inflammatory properties and it maintains relatively low off-target cytotoxicity. In our paper, we present the fabrication, characterization and cytocompatibility of titanium oxide (TiO_2_) nanotube surface decorated with various surface densities of chemically synthesized selenium nanoparticles. To evaluate antibacterial and anti-cancer properties of such nanostructured surface, gram negative bacteria *E*. *coli*, cancerous osteoblast like MG-63 cells and non-cancerous fibroblast NIH/3T3 were cultured on designed surfaces. Our results suggested that selenium nanoparticles improved antibacterial properties of titanium dioxide nanotubes and confirmed the anticancer activity towards MG-63 cells, with increasing surface density of nanoparticles. Further, the selenium decorated TiO_2_ nanotubes suggested deteriorating effect on the cell adhesion and viability of non-cancerous NIH/3T3 cells. Thus, we demonstrated that selenium nanoparticles decorated TiO_2_ nanotubes synthesized using sodium selenite and glutathione can be used to control bacterial infections and prevent the growth of cancerous cells. However, the higher surface density of nanoparticles adsorbed on the surface was found to be cytotoxic for non-cancerous NIH/3T3 cells and thus it might complicate the integration of biomaterial into the host tissue. Therefore, an optimal surface density of selenium nanoparticles must be found to effectively kill bacteria and cancer cells, while remaining favorable for normal cells.

## Introduction

The widespread use and persisting negative properties of some metallic biomaterials for tissue engineering or surgical instruments aimed researches to modulate the surface characteristics of biomaterials into the form with desired functional surface properties. The rapid progress in nanotechnology enabled us to approach and mimic the tissue environment via surface modifications including chemistry, topography or roughness that can significantly change the interface between individual material and tissue cells or bacteria and thus, results in different responses in cell adhesion, viability, metabolism, antibacterial or anti-inflammatory activity. For instance, it was previously reported in independent *in vitro* studies that the nanotopography plays a crucial role in the character and strength of the cellular interaction to such a modified surface with a positive impact on the resulting cellular responses [1–5]. Moreover, nanostructured biomaterials were considered to exhibit various qualities, such as antibacterial activity [6, 7] or improved bioactivity [8, 9] that were not observed for their non-structured forms.

Materials such as titanium (Ti) and its alloys are widely used in many replacements including orthopaedical, dental and cardiovascular implants and medical devices [10]_._ The favorable mechanical properties, exceptional corrosion resistance and biocompatibility of titanium [11] were attributed to a passive thin film of titanium dioxide (TiO_2_) formed on Ti surface [8]. This thin layer was also considered to impart bioactivity and chemical bonding between implant and the bone [2]. Nanostructuring of titanium film into the form of nanotubes (TNTs) has attracted great attention for improving cell adhesion, growth and differentiation [9, 12, 13]. Later findings proved the strong relation between the cell responses and nanotube dimensions [4, 14, 15]. For example, mesenchymal stem cells showed the improved response on nanotubes with smaller diameters (~15 nm) [4, 16], while osteoblasts preferred nanotubes with bigger diameters (~70 nm) [12]. The adhesion was faster and stronger compared to the control, which was thought to avoid formation of fibrous tissue caused by a weak cell-surface interaction [2].

Many approaches were subsequently introduced to enhance the performance of TNT surfaces. Some aimed to increase the antibacterial activity through loading the nanotubes with various antibiotics, such as vancomycin [17] and gentamicin [18] or their decoration/doping with various nanoparticles, including gold [19, 20], silver [8, 21] and zinc oxide [22]. Other addressed the biocompatibility through coating with bioactive compounds, such as PLGA [23], chitosan [23], hydroxyapatite [24] or growth factors [25].

Recently, treatment of bio-surfaces with selenium attracted attention. Selenium is an important element that plays crucial role in preventing cancer and protecting cells from oxidative damage [26, 27]. It has been reported to exhibit not only antibacterial [28–31], but also anti-cancer [28, 32] and anti-inflammatory [29, 33] properties that make selenium interesting for various applications including oncology [28, 32], regenerative medicine [34] and tissue engineering [29, 30, 35–37]. Selenium nanoparticle (SeNPs) decorated TNTs were studied and reported previously for its antibacterial and anti-inflammatory properties [29]. Results suggested that selenium nanoparticles enhanced antibacterial properties of TNTs which caused the decrease in colony forming units of both gram-positive and gram-negative bacteria. How such a surface effected cancerous and non-cancerous cells remained unanswered. In another reported research [28], the release of selenium nanoparticles from TiO_2_ nanotubes covered with chitosan was studied for antibacterial activity. Authors declared that such a fabricated and designed surface positively influenced the viability of osteoblasts and negatively affected cancerous cells, while promoted the antibacterial activity of TNTs. However, this antibacterial effect could be attributed to chitosan, which is, in the fact, antimicrobial [38] and biocompatible [39]. Authors in this study also admitted, that no discernable layer of Se or Se nanoparticles was observed on their samples.

In our work, we introduced the fabrication of TiO_2_ nanotubes of 50 nm in diameter via anodic oxidation of titanium layer deposited on silicon wafer. Nanotubes were decorated with chemically synthetized selenium nanoparticles of three different surface densities. Designed films were characterized with contact angle measurement, SEM, XPS and AFM techniques. Due to the absence of reports regarding the interaction of such a surface with tissue cells, we studied mutual effect of selenium nanoparticles and TiO_2_ nanotubes on antibacterial properties against *E*. *coli* and viability of osteosarcoma MG-63 cells and non-cancerous NIH/3T3 fibroblasts.

## Materials and methods

### Preparation of nanotubes via anodic oxidation

**Fig 1:**
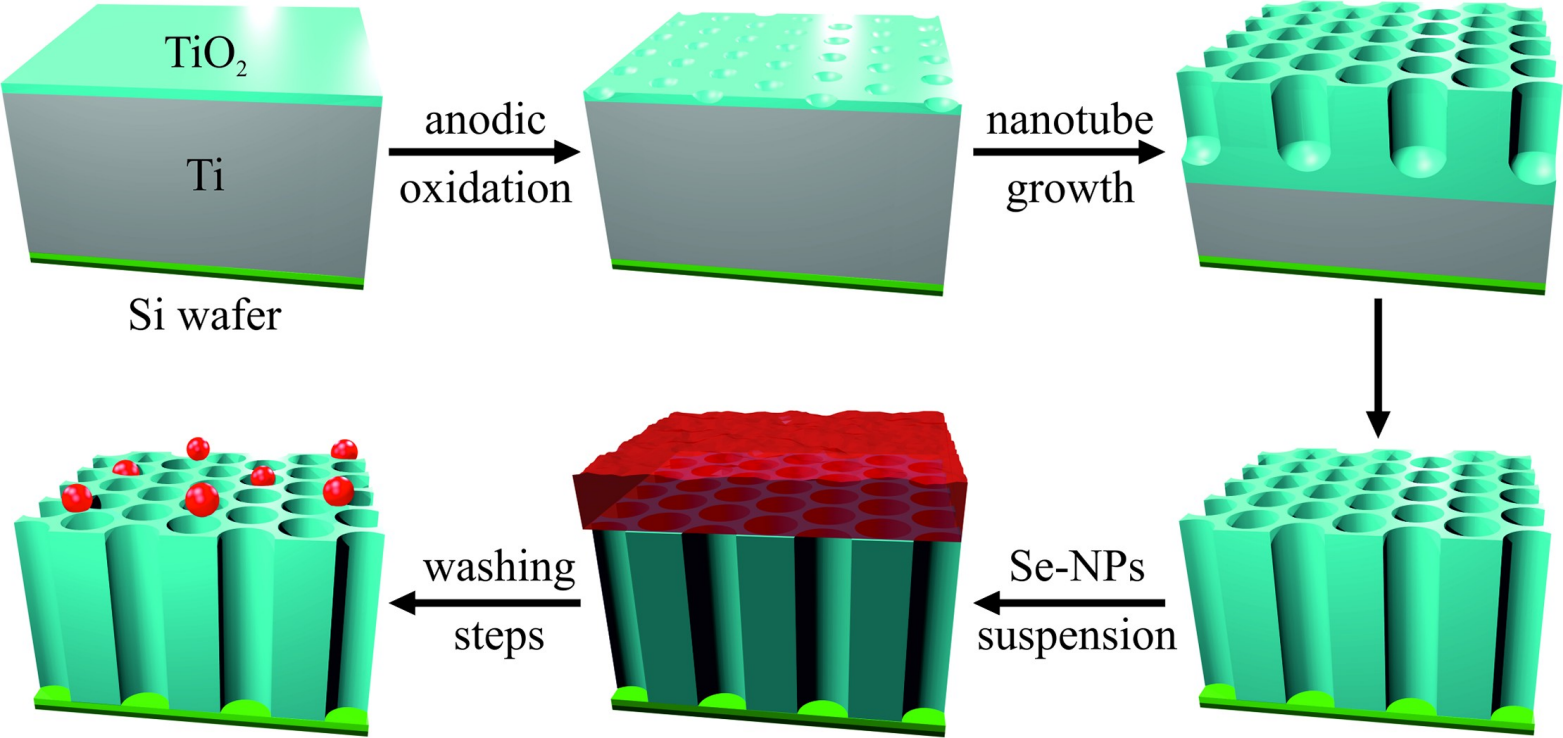
Fabrication scheme of Se-NP decorated TiO_2_ nanotube film. TiO_2_ nanotubes were fabricated via anodic oxidation of 500 nm thick titanium layer deposited on silicon wafer. Nanotubes were decorated with different concentrations of selenium nanoparticles.

TiO_2_ nanotubes were fabricated from 500 nm thick titanium layer sputter-deposited on silicon wafer via electrochemical anodization according to protocol established previously [40]. The fabrication process is schematically depicted in Fig 1. Briefly, electrochemical anodization was performed with voltage ramp from 0 V to 15 V and the step of 1 volt per second in the electrolyte solution of ethylene glycol (C_2_H_6_O_2_, p.a., Penta, CZ), 1.2 wt% ammonium fluoride (NH_4_F, Sigma Aldrich, DE) and 2 vol% of deionized water (Millipore Corp., USA, 18,2 MΩ). When the voltage reached the maximum set value, the voltage was maintained until the current reached zero value. Samples were then rinsed with deionized water, dried with stream of nitrogen and subsequently annealed in vacuum furnace at 450°C for 3 hours, with the heating ramp of 5°C per minute.

#### Decoration with selenium nanoparticles

The protocol for preparation of selenium nanoparticle solution was inspired by previous study carried by Liu et al. [29]. The 3 mL of 100 mmol L^-1^ L-glutathione (Sigma-Aldrich) and 3 mL of 100 mmol L^-1^ sodium selenite (Sigma-Aldrich) were blended together. To control the amount of selenium nanoparticles decorated onto the surface, different volumes of ultrapure water were added to individual mixtures– 9 mL for low, 6 mL for medium and 3 mL for high selenium nanoparticle concentration. Finally, 50 μL of 1 mol L^-1^ NaOH solution was added to initiate the reaction at room temperature. The formation of nanoparticles was optically observed as a gradual change in the color from transparent to red. 100 μL of nanoparticle suspension was then introduced onto the nanostructured surface and incubated for 20 minutes. After that, samples were rinsed several times, and subsequently soaked in ultrapure water to remove non-adsorbed particles. Scanning electron microscopy and atomic force microscopy was used to check the quality of nanoparticle decoration, and to analyze the amount and diameter of selenium nanoparticles. Prior to bio-characterizations, fabricated surfaces were sterilized with UV irradiation for 15 minutes.

### Characterization of nanostructured film

The wettability of samples was measured at room temperature by determination of water contact angle of 5 μL water drop introduced on sample surface after UV irradiation (Phoenix 300, Surface & Electro Optics Co.,Ltd, KOR). Contact angle was evaluated using tangent line method 2 (higher angles) and trigoniometric functions method (lower angles) implemented in software Surfaceware 8. The surface morphology and nanoparticles size was characterized with scanning electron microscope (SEM, Mira II, Tescan, CZ) and the roughness was calculated by atomic force microscopy in dry non-contact mode (AFM, NanoWizzard3, JPK). Chemical composition of samples was determined with X-ray photoelectron spectroscopy (XPS, AXIS Supra, Kratos Analytical Ltd, UK). The XPS spectra were analyzed by a peak fitting software (CasaXPS version 2.3.18PR1.0) provided by SPECS GmbH (Berlin, Germany). Raw data were processed by the subtraction of a Shirley background for secondary electrons and element peak fitting was used to estimate the relative element molar fraction. The integral of the peak was divided by a relative sensitivity factor (R.S.F.), which is characteristic for each element. Finally, the concentration profile of selenium released from the surface was determined with inductively coupled plasma mass spectrometry (ICP-MS). Decorated samples were immersed into 3 mL of ultrapure water for 15 days. At selected time points, 1.5 mL of solution was collected. Samples were subsequently dried with nitrogen stream and immersed into 3 mL of fresh ultrapure water.

### Antibacterial test

Antibacterial properties of selenium NPs decorated nanotubes were evaluated through the bacterial viability assay with gram negative *E*. *coli*. Non-decorated nanotube surface was used as a control. The samples were rinsed with water and sterilized with UV for 15 minutes before each experiment. Briefly, 50 μL of bacterial suspension was spread onto the surface and incubated 4 hours at 37°C. Subsequently, adhered bacteria were washed out with phosphate buffer saline (PBS) and collected bacterial suspension was diluted 100 times with PBS. Bacteria cultured plates were prepared using agar, yeast extract, NaCl and tryptone. The 200 μL of diluted solution was inoculated on prepared agar plates and incubated for 24 hours at 37°C and 80% humidity. Agar plates were then photographed and colony forming units were calculated (CFU mL^-1^).

### Cell culture

The cancerous MG-63 cells and non-cancerous NIH/3T3 cells were maintained in complete Dulbecco's Modified Eagle's medium (DMEM) supplemented with 10% fetal bovine serum (FBS), 2 mmol L^-1^ L-glutamine, and 5% penicillin/streptomycin (50 U mL^-1^ and 50 μg mL^-1^) at 37°C in a humidified 5% CO_2_ incubator. Cells were harvested by trypsinization with 0.25% trypsin-EDTA solution at 75% confluency and seeded with a defined density onto the sterile samples placed in a polystyrene microplate.

### Cell adhesion and viability

The adhesion of MG-63 and NIH/3T3 cells onto TiO_2_ nanotubes decorated with selenium NPs and onto the control (TiO_2_ nanotubes without selenium nanoparticles) was qualitatively evaluated from images taken by an optical microscope (Axio Imager M2m, Zeiss). TiO_2_ and TiO_2_ –SeNPs samples were placed in a 24-well plate and used as the surface for cell seeding at a density of 1∙10^3^ cells per area. The images of cells were taken at 3 hours. Viability of MG-63 cells was measured with XTT assay and evaluated on days 1, 2, and 6 after the seeding; the initial cell density was 1∙10^3^ cells per area. Briefly, the cells were incubated for a defined period of time and then gently washed twice with pre-heated phosphate buffer saline (PBS). The mixture of 100 μL culture medium and 50 μL of tetrazolium dye (XTT, 1 mg mL^-1^ in DMEM and 25 μmol L^-1^ PMS in PBS) was added into each well containing the samples and incubated 2 hours in CO_2_ incubator. 100 μL solution were transferred from each well into a new 96-well plate and the absorbance was measured at 450 nm with a microplate spectrophotometer (Beckman Coulter Paradigm). The live/dead staining of MG-63 cells and 3T3 fibroblasts was performed at the day 6. The cells were gently rinsed with pre-warmed PBS and incubated 15 min with 2 μM Calcein-AM and 1.5 μM propidium iodide solution in PBS. The cells were finally rinsed twice in PBS before imaging with a fluorescent microscope coupled to a CCD camera (Zeiss, Germany).

### Statistical analysis

Mean values and standard deviations of obtained data were calculated. Statistically significant differences (p < 0.05) were confirmed using Student’s t-test. All shown data are expressed as the mean ± standard deviation.

## Results and discussion

### Surface characterization

**Fig 2:**
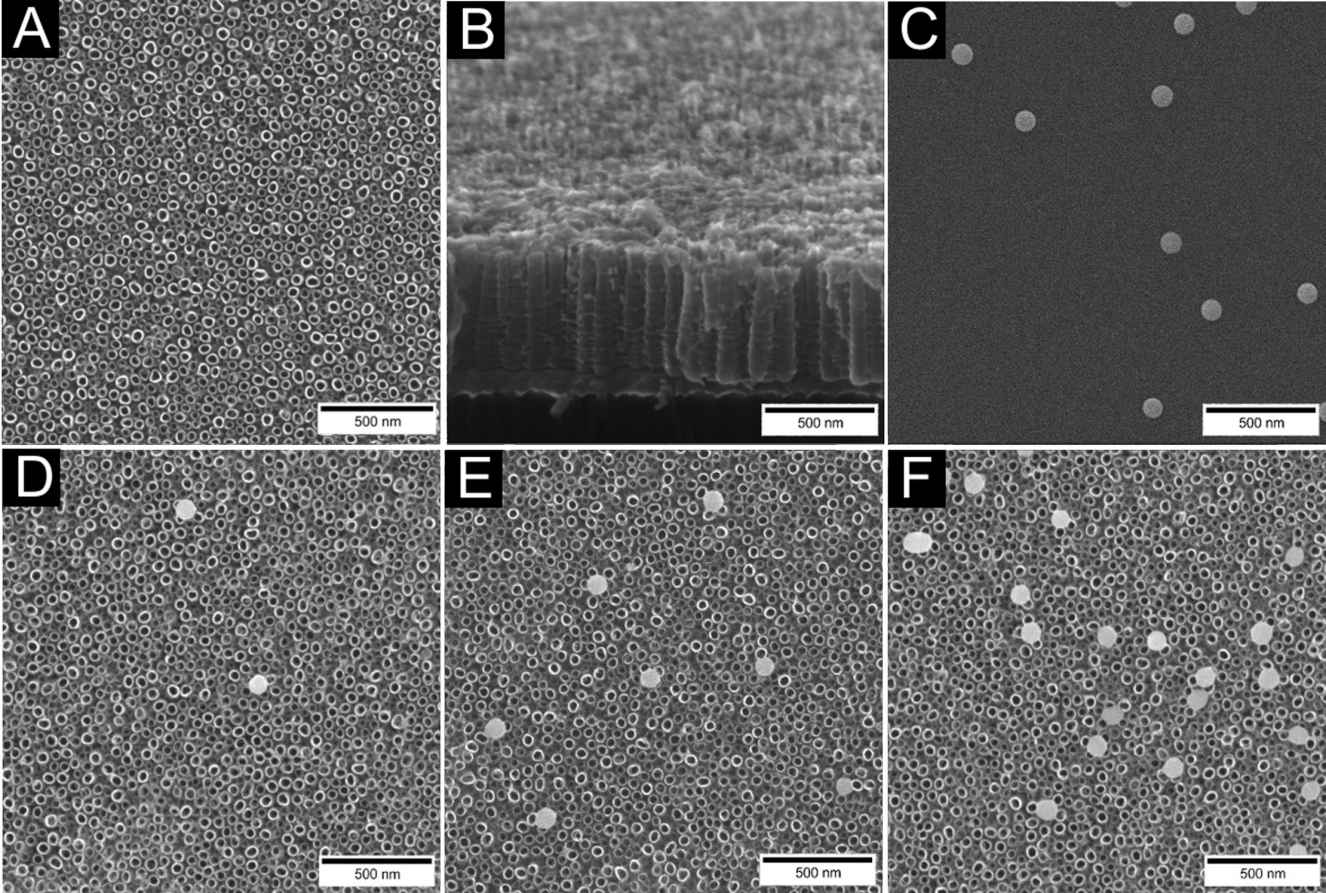
SEM images of fabricated nanostructures. Annealed TiO_2_ nanotubes (A), their cross section (B), and selenium nanoparticles (C). TiO_2_ nanotubes decorated with Se-Low (D), Se-Medium (E) and Se-High (F) concentrations of nanoparticles.

**Fig 3:**
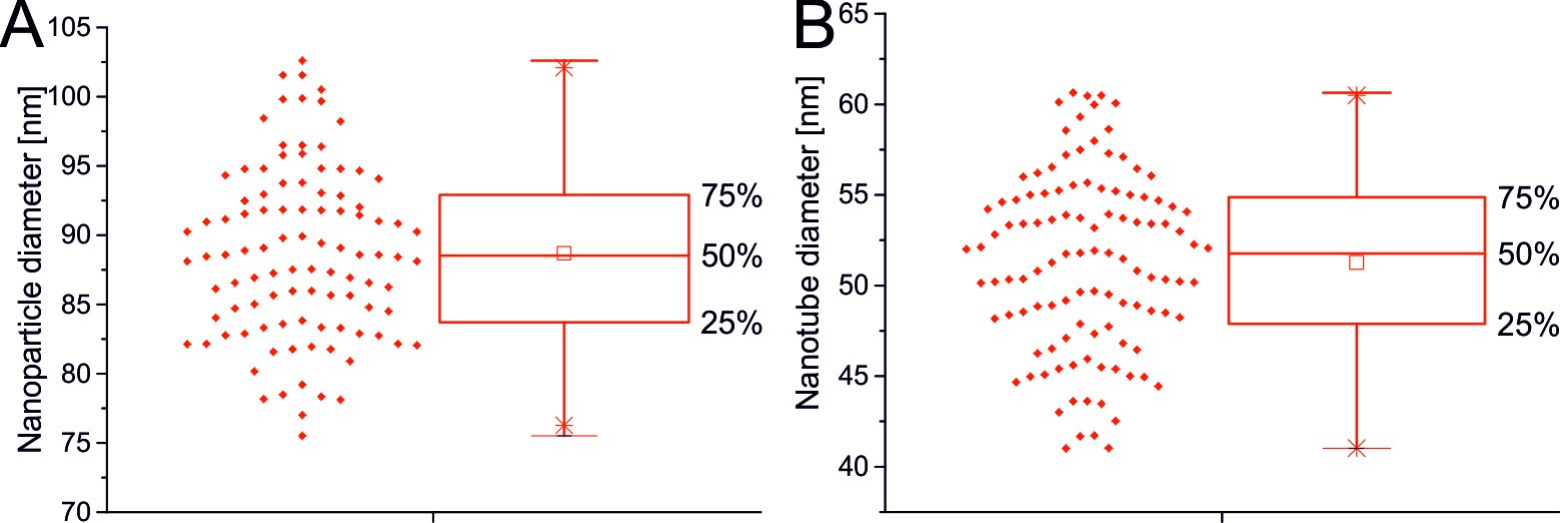
**Boxplot of normal size distributions of nanoparticle (A) and nanotube (B).** Both the boxplot and Shapiro-Wilk test of normality suggested normal distributions.

Selenium NPs decorated TiO_2_ nanotubes were fabricated via anodic oxidation of titanium layer deposited on silicon wafer followed by the adsorption of chemically synthesized selenium nanoparticles. Fabrication of nanostructured surface resulted in TNTs with the diameter of 51.72 ± 5.55 nm (Fig 2A) and length of 500 nm (Fig 2B). The surface was decorated with spherical selenium nanoparticles (Fig 2C) of 88.93 ± 6.87 nm in size, which is significantly bigger than nanotube diameter. Thus the nanoparticles covered the top of the nanotubes at Se-Low (Fig 2D), Se-Medium (Fig 2E) and Se-High (Fig 2F) densities corresponding to number of 3.2 ± 1.14 (low), 9.1 ± 1.20 (medium) and 18.5 ± 2.37 (high) particles per 4 μm^2^, respectively. According to Boxplot of obtained data (Fig 3) and Shapiro-Wilk test of normality, both nanoparticle and nanotube size have normal distributions

SEM analysis also revealed the discrete TiO_2_ nanotubes with the nanoparticle density and nanotube diameter consistent over the whole surface of the sample. AFM analysis further showed the increase in root mean squared (RMS) roughness of samples with increasing surface density of nanoparticles. The RMS value from bare TNTs to Se-High TNTs ranged from 16.6 nm to 24.1 nm.

Measurement of UV-treated TNTs wettability showed significant difference between decorated and non-decorated TiO_2_ nanotubes. The contact angle of bare nanotubes and nanoparticle decorated nanotubes was assessed to values ~20° and ~48°, respectively. The difference between individual nanoparticle surface densities was ~1.5°. Selenium nanoparticles can slightly increase the contact angle of TiO_2_ surfaces as was previously reported [41] and this effect was also retained after the UV irradiation.

X-ray photoelectron spectroscopy of selenium decorated TiO_2_ nanotubes was done to confirm the presence selenium in the samples. There was no significant change in bonding states of Ti2p and O1s after the decoration with nanoparticles. The XPS quantitative analysis of selected elements was summarized in Table 1. The percentage of selenium Se3d proportionally increased with increasing surface density of nanoparticles. We also observed a peak corresponding to the presence of sulfur. The sulfur peak was observed for all nanoparticle decorated samples even after multiple washing steps and 24 hours immersion in ultrapure water. It could correspond with the adsorption of glutathione, or other product of reaction containing sulfur on the fabricated surface.

**Table 1:** XPS analysis of selenium decorated TiO_2_ nanotubes. Relative percentage of selected elements calculated from narrow spectra.

	TNTs [%]	Se-Low [%]	Se-Medium [%]	Se-High [%]
O1s	55,93	56,12	55,89	51,92
Ti2p	12,75	11,69	11,98	11,11
C1s	31,31	32,19	31,76	35,24
Se3d	0,00	0,08	0,46	1,73

**Fig 4:**
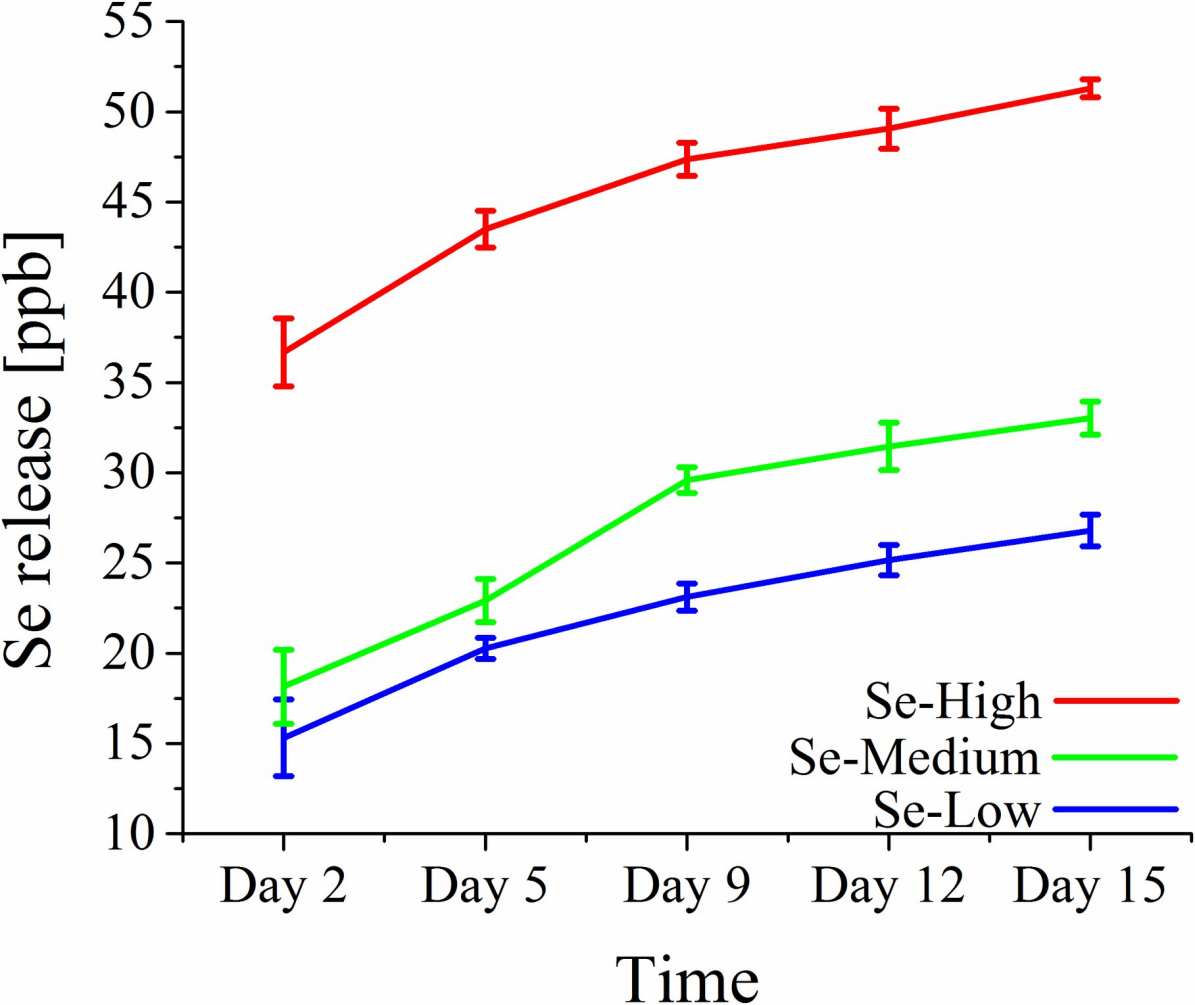
The cumulative release profile of selenium. Individual samples were measured with ICP-MS technique. Measured release was highly dependent on initial SeNPs concentrations.

The ICP-MS analysis introduce powerful and sensitive technique capable of detecting metals and several non-metals at concentrations as low as one part in 10^15^ (part per quadrillion, ppq). We used this technique to obtain the release profile of selenium from the nanotube surface. The analysis showed the minimal release rate of selenium at maximum tens of ppb (Fig 4), suggesting a strong bond between SeNPs and nanotubes. The biggest contribution to the total release was during the first 24 hours, which can be still attributed to the washing selenium out from the nanotubes. Over period of time, the released amount of selenium steadily decreased. Samples were then checked with SEM microscope. Compared to images taken before the measurement, no significant difference in SeNP surface density was found. Further, the release rate was significantly lower than in research done by Liu et al. [29], in which authors attributed the importance of release to the cytotoxicity of selenium. The question is whether such a low concentration of selenium can result in the toxicity for some bacteria and tissue cells. Taking into the consideration it beneficial properties in the human body at higher concentration than our measurable ppb values, we believe that there is another mechanism of toxicity via the interaction of cell membrane with combination of nanoparticle and nanotube properties, nanoparticle size and surface density.

### Antibacterial properties of Se NPs—TiO_2_ nanotubes

**Fig 5:**
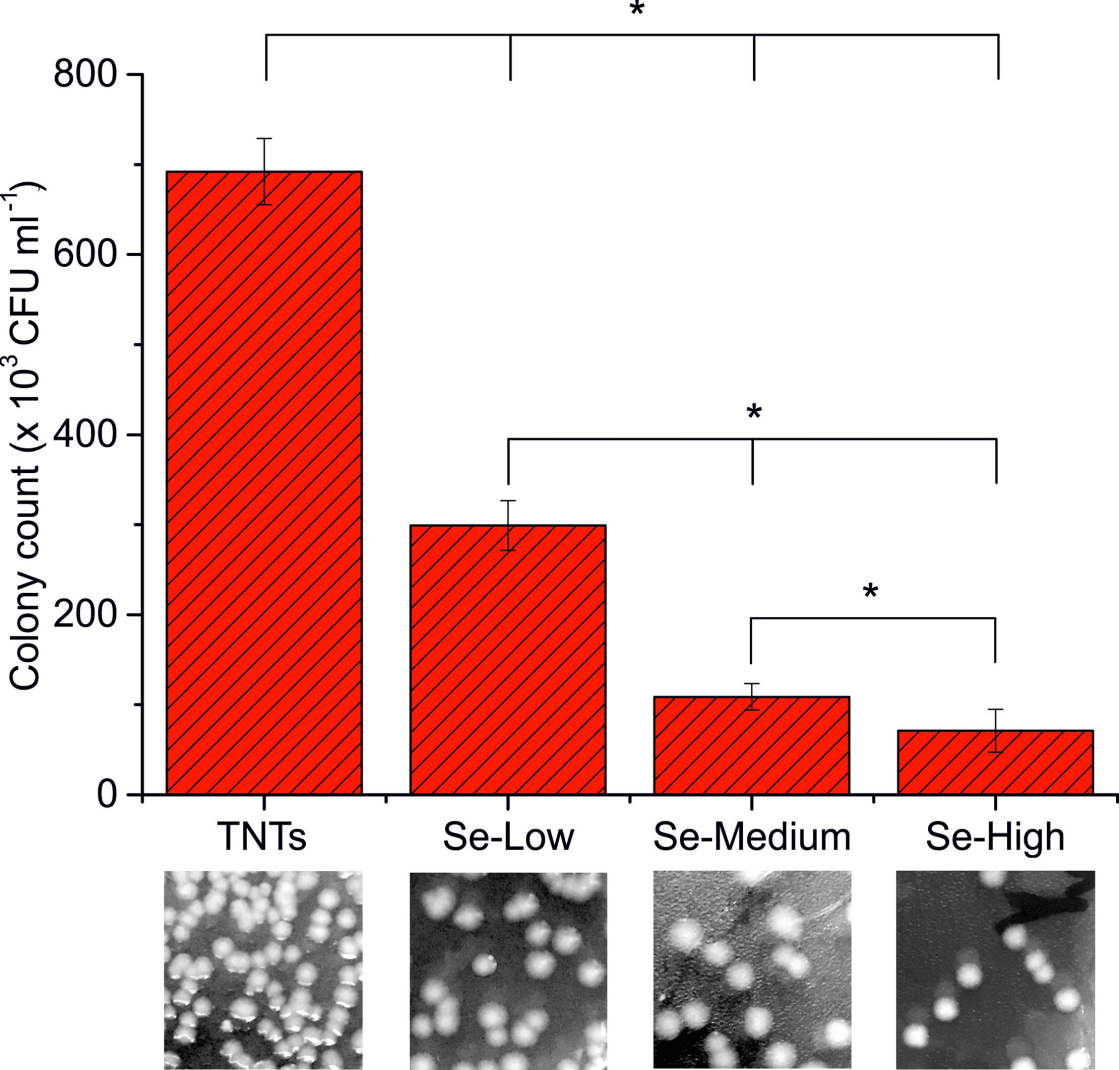
Bacterial assay of viability expressed in colony forming units. Se nanoparticle decorated TiO_2_ -TNTs were exposed to gram negative bacteria *E*. *coli* for 4 hours and the antibacterial effect Se-TNTs was compared with undecorated TNTs. * indicates significant difference between compared samples (p = 0.05).

To protect the adhesion and proliferation of pathogenic bacteria on biomaterials consist in coatings with antibiotics, biocidal agents or antibacterial nanoparticles. Selenium nanoparticles are well-known for antibacterial properties to the wide range of bacteria even if the mechanism of action is still unknown [42]. It has been reported by Tran et al. [30] that selenium nanoparticles inhibited the growth of gram-positive *Staphylococcus aureus*. Liu et al. [29] incorporated these nanoparticles into TNTs to enhance the antibacterial activity of TNT surface to *Escherichia coli* with 50% efficiency compared to bare TNTs in 24 hours. In our experiment, we compared the antibacterial efficiency of selenium decorated TiO_2_ nanotubes at different selenium nanoparticle surface density against *E*. *coli* in a short four-hour incubation. Compared to the bare nanotubes, selenium decorated TNTs exhibited enhanced antibacterial properties (Fig 5). Short time interaction of surface with bacteria was efficient to decrease the number of *E*. *coli* colonies for Se-Low and Se-High about 60% and 90%, respectively. Even if we confirmed the antibacterial properties of Se-TNTs surface, the question is whether bacteria were killed by selenium release in ppb concentration, as indicated by ICP-MS at the first 24 hours or by the nanoscale surface topography and chemistry of Se-TiO_2_-TNTs which can be non-adhesive, anti-proliferative or membrane damaging for some bacteria [29, 30, 36, 43].Selenium nanoparticles and TiO_2_ are negatively charged at physiological pH and thus repulsive for the negatively charged membrane of *E*. *coli* [44]. This might also suggest the inhibition mechanism of *E*. *coli* adhesion and proliferation on Se-TiO_2_-TNTs surface at a short time incubation.

### Viability assay of normal and cancer cells

**Fig 6:**
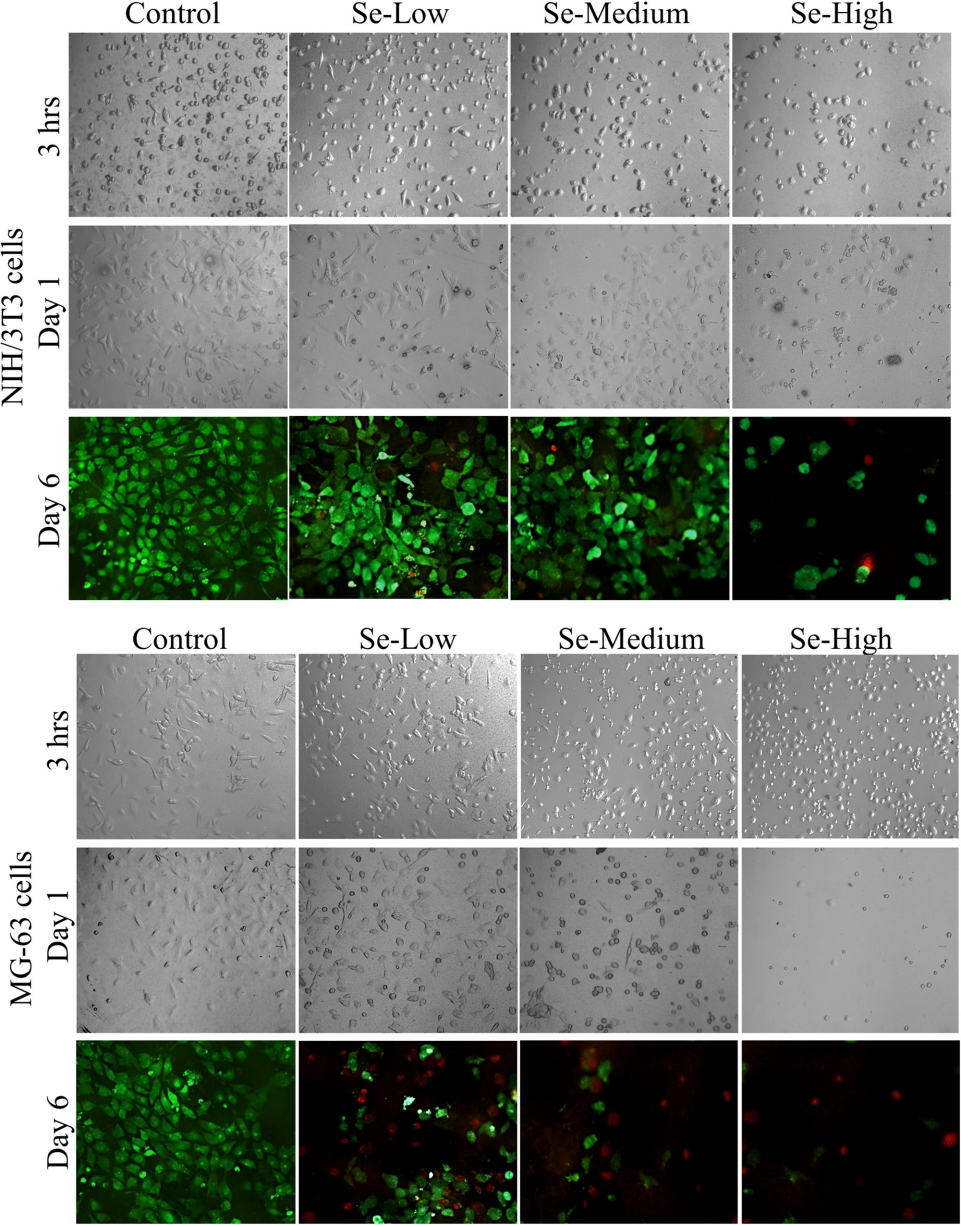
Adhesion and morphology of MG-63 (cancer) and NIH/3T3 (normal) cells cultured on Se-NPs decorated TiO_2_ nanotubes. DIC images were taken after 3 and 24 hours cultivation. Live/dead staining of cells was performed with calcein (green color) and propidium iodide (red color) at the day 6.

**Fig 7:**
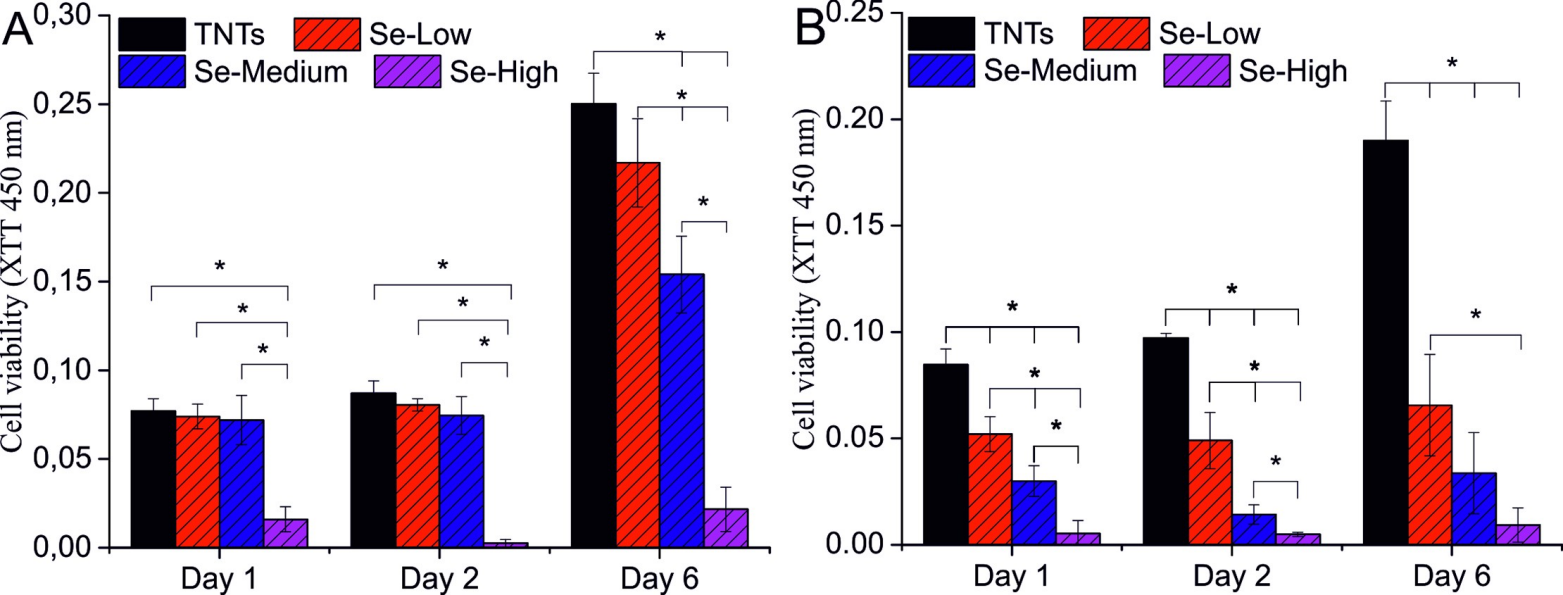
**XTT viability assay of MG-63 (B) and NIH/3T3 (A) cells cultured on Se-NPs decorated TiO**_**2**_ **nanotubes.** * indicates significant difference between compared samples (p = 0.05).

Adhesion plays a fundamental role in cell development, differentiation, communication and migration, and during the pathogenesis of a wide range of diseases including cancer [45], osteoporosis [46], and atherosclerosis [47]. The strength of cell adhesion to substrate is a crucial consideration in biomaterial design and development. On the other hand, selenium is known to be involved in anti-cancer activity while protecting the benign cells [27, 28, 32, 48]. Thus we studied the effect of selenium nanoparticle decorated TiO_2_ nanotubes on adhesion and proliferation of cancer (MG-63 cells) and normal (NIH/3T3 cells) cells. The adhesion and morphology of both cells was qualitatively evaluated by taking DIC images 3 and 24 hours after cell seeding as shown in Fig 6. The NIH/3T3 cells adhered on control TNTs and Se-Low TNTs surface with fibroblastic well spread elongated morphology. Se-Medium surface showed a little deterioration in cells morphology turning the elongated morphology to the rounded shape but still well adhered. The NIH/3T3 cells on Se-High surface were completely round in shape with obvious apoptotic cells. The unfavorable conditions reflecting the change in cell morphology resulted in lower number of attached cells. The similar effect of selenium decorated nanotubes was observed for adhesion and morphology of cancerous MG-63 cells (Fig 6). These cells at the day 1 attached to the control and Se-Low surfaces but exhibited more rounded shape compared to the noncancerous cells (Fig 6). Cell adhesion experiment demonstrated that the selenium nanoparticle decorated TiO_2_ nanotubes caused unfavorable conditions for cancer MG-63 and normal NIH/3T3 cells, particularly at Se-Medium and Se-High TNTs surface, compared to the control TNTs and Se-Low surface. In order to know whether these microscopic observations were not done by the slow adhesion rate, we performed proliferation assay by measurement of cellular activity using XTT assay. Our results with NIH/3T3 cells suggested that there was no significant difference in proliferation rate at day 1 and 2 for Se-Low and Se-Medium TNTs surfaces compared to the control group (Fig 7A). At the day 6, XTT assay showed increased proliferation rate of NIH/3T3 cells. The decreasing tendency of measured values at this day towards Se-Medium can be attributed to the slower adhesion, activity or proliferation of cells on such surfaces. Anyway, the Se-Low and Se-Medium surface promoted activity and proliferation of NIH/3T3 cells. Proliferation rate in NIH/3T3 cells cultured on Se-High TNTs surface was significantly decreased at day 1, 2 and 6. It came out from the smaller initial number of cells adhered well on Se-High surface as observed with adhesion assay and suggests that this selenium nanoparticle surface density was not compatible with the adhesion and proliferation of noncancerous NIH/3T3 cells compared to the Se-lower surface densities and control TNTs surfaces. The similar results were obtained for the cancerous MG-63 cells (Fig 7B). The unfavorable effect of selenium nanoparticle density for cancerous cells was stronger at Se-Medium surface compared to the noncancerous cells and maximal at Se-High surface. We can also observe from our results that MG-63 cells did not adhere well selenium decorated surfaces but when adhered, they did not increase activity or proliferation rate since no significant change in absorbance was observed at day 6 compared to day 1 and 2. Finally, we did live/dead staining of both cells to check the viability of cells at the day 6. The images (Fig 6) confirmed our quantitative results obtained from the proliferation test. The cancerous cells gradually detached the selenium decorated surface and compared with the control, mostly dying cells occurred attached the surface (Fig 6, red color). Fibroblasts showed significant decrease in a number of adhered cells on Se-High surface and a few dying cells on Se-Medium surface which again supported our results from proliferation assay.

Our observation is consistent with other similar report of Chen et al. [28] in which the selenium nanoparticles released from TiO_2_ nanotubes and diffused through the chitosan layer, decreased adhesion and viability of cancerous osteoblasts. Other reports suggesting selenium as a promising and favorable for noncancerous cells [28, 32] was not confirmed in our experiments since the Se-High TNTs surface was significantly suppressing the noncancerous NIH/3T3 cells adhesion and viability. To conclude these results, the exact mechanism that makes the anti-cancer action of selenium work is still a subject of research as well as much work must be done regarding the influence of selenium towards noncancerous cells. However, selenium nanoparticle decorated TiO_2_ nanotubes can be promising biomaterial surface for tissue engineering since the mechanisms of action combining the antibacterial selenium nanoparticles and nanostructured TiO_2_ surface is still unknown.

## Conclusion

In this paper, TiO_2_ nanotubes with 51,72 ± 5,55 nm diameter decorated with different surface densities of spherical selenium nanoparticles with 88,93 ± 6,87 nm diameter were fabricated via anodic oxidation and characterized with SEM, XPS and AFM. The release rate of selenium measured with ICP-MS was found out very low for a long period of time, which indicates for the strong and stable adsorption of selenium nanoparticles on TiO_2_ nanotubes. The antibacterial activity of Se-NPs decorated TNTs was significantly enhanced in 4 hours incubation for gram negative *E*. *coli* which suggest the strong antibacterial effect of selenium compared to the bare TNTs surface. The cell adhesion and proliferation of cancerous MG-63 cells was obviously decreased on selenium decorated surface, confirming the anti-cancer activity of selenium compared to the bare TiO_2_ nanotubes. The NIH/3T3 fibroblasts adhered and proliferated on bare, Se-Low and Se-Medium TNTs surfaces. The Se-High surface density of nanoparticles was found to be incompatible with NIH/3T3 cell adhesion and proliferation. Therefore, it is important and desirable to find an optimal surface density of selenium nanoparticles to be decorated on TiO_2_ nanotubes including the nanoparticle and nanotube diameters that effectively kill bacteria, cancer cells and remains favorable to the normal cells.

## Supporting information

S1 File Individual datasets. File contains raw data from which the graphs were drawn.
(DOCX)
