# Supplementary material for: Enhanced antibacterial and anticancer properties of Se-NPs decorated TiO2 nanotube film
Source: PLoS One. 2019 Mar 22;14(3):e0214066. doi: 10.1371/journal.pone.0214066 (PMC6430414; doi:10.1371/journal.pone.0214066)

Supporting information

# Dataset – SeNP diameter [nm]

| 102,610 | 92,856 | 88,465 | 83,58 |
| --- | --- | --- | --- |
| 99,883 | 92,475 | 88,427 | 83,333 |
| 101,562 | 92,034 | 88,119 | 83,329 |
| 101,562 | 91,844 | 88,119 | 83,289 |
| 100,522 | 91,842 | 87,540 | 82,890 |
| 99,811 | 91,797 | 87,526 | 82,885 |
| 99,675 | 91,797 | 87,346 | 82,762 |
| 98,443 | 91,739 | 87,264 | 82,737 |
| 98,221 | 91,547 | 86,936 | 82,147 |
| 96,495 | 91,443 | 86,918 | 82,147 |
| 96,495 | 91,146 | 86,567 | 82,124 |
| 96,389 | 91,006 | 86,567 | 82,054 |
| 95,882 | 90,960 | 86,253 | 81,949 |
| 95,783 | 90,848 | 86,137 | 81,772 |
| 94,822 | 90,249 | 85,987 | 81,772 |
| 94,807 | 90,249 | 85,960 | 81,575 |
| 94,793 | 89,910 | 85,661 | 80,897 |
| 94,772 | 89,797 | 85,661 | 80,173 |
| 94,650 | 89,424 | 85,637 | 79,203 |
| 94,327 | 89,076 | 85,028 | 78,478 |
| 94,076 | 89,076 | 84,786 | 78,344 |
| 93,786 | 88,886 | 84,705 | 78,168 |
| 93,755 | 88,580 | 84,505 | 78,125 |
| 93,060 | 88,580 | 84,035 | 77,016 |
| 92,958 | 88,580 | 83,830 | 75,521 |

| N | Mean | Standard Deviation |
| --- | --- | --- |
| 100 | 88,93469 | 6,86794 |

# Dataset – TNT diameter [nm]

| 60,461 | 55,247 | 52,12 | 48,898 | 44,433 |
| --- | --- | --- | --- | --- |
| 53,179 | 55,21 | 52,061 | 48,898 | 43,625 |
| 47,331 | 55,074 | 52,008 | 48,845 | 43,625 |
| 53,731 | 55 | 51,928 | 48,615 | 43,468 |
| 60,634 | 55 | 51,795 | 48,539 | 43,013 |
| 60,491 | 54,872 | 51,794 | 48,497 | 42,518 |
| 60,122 | 54,722 | 51,745 | 48,392 | 41,712 |
| 60,051 | 54,688 | 51,482 | 48,247 | 41,672 |
| 59,984 | 54,598 | 51,27 | 48,181 | 41,021 |
| 59,298 | 54,353 | 50,812 | 47,891 | 41,016 |
| 58,626 | 54,205 | 50,805 | 47,744 |  |
| 58,558 | 54,072 | 50,444 | 47,096 |  |
| 57,976 | 53,937 | 50,371 | 46,806 |  |
| 57,502 | 53,883 | 50,337 | 46,516 |  |
| 57,295 | 53,713 | 50,337 | 46,451 |  |
| 57,203 | 53,631 | 50,215 | 46,265 |  |
| 57,092 | 53,485 | 50,198 | 45,948 |  |
| 56,532 | 53,449 | 50,168 | 45,592 |  |
| 56,446 | 53,409 | 50,147 | 45,489 |  |
| 56,205 | 53,401 | 49,689 | 45,41 |  |
| 56,05 | 53,401 | 49,633 | 45,391 |  |
| 56,005 | 53,321 | 49,518 | 45,091 |  |
| 55,683 | 52,991 | 49,178 | 44,997 |  |
| 55,526 | 52,811 | 49,04 | 44,964 |  |
| 55,354 | 52,266 | 48,898 | 44,955 |  |

| N | Mean | Standard Deviation |
| --- | --- | --- |
| 110 | 51,71687 | 5,55478 |

# Dataset – SeNP densities [particles per 4µm^2^]

| Low | Medium | High |
| --- | --- | --- |
| 4 | 9 | 17 |
| 4 | 10 | 16 |
| 1 | 8 | 20 |
| 4 | 7 | 17 |
| 2 | 10 | 24 |
| 4 | 9 | 17 |
| 3 | 11 | 19 |
| 4 | 9 | 20 |
| 4 | 10 | 17 |
| 2 | 8 | 18 |

|  | N | Mean | Standard deviation |
| --- | --- | --- | --- |
| Low | 10 | 3,2 | 1,13529 |
| Medium | 10 | 9,1 | 1,19722 |
| High | 10 | 18,5 | 2,36878 |

# Dataset-AFM

|  | Roughness Rms [nm] |
| --- | --- |
| Control | 16,6 |
| Low | 17,7 |
| Medium | 18,1 |
| High | 24,1 |

# Dataset XPS

## C1s

| Binding energy [eV] | TNTs | Se-Low | Se-Medium | Se-High |
| --- | --- | --- | --- | --- |
| 294 | 228,667 | 268 | 306,667 | 463,333 |
| 293,95 | 219,333 | 261,333 | 332,667 | 465,333 |
| 293,9 | 240,667 | 266,667 | 348 | 474,667 |
| 293,85 | 231,333 | 249,333 | 314,667 | 432 |
| 293,8 | 256,667 | 251,333 | 317,333 | 475,333 |
| 293,75 | 233,333 | 248,667 | 328 | 466,667 |
| 293,7 | 256,667 | 262 | 334,667 | 445,333 |
| 293,65 | 248 | 253,333 | 353,333 | 464 |
| 293,6 | 232 | 259,333 | 341,333 | 462 |
| 293,55 | 236,667 | 256 | 314 | 469,333 |
| 293,5 | 233,333 | 255,333 | 333,333 | 470 |
| 293,45 | 242,667 | 245,333 | 304,667 | 453,333 |
| 293,4 | 243,333 | 260,667 | 298 | 438 |
| 293,35 | 225,333 | 267,333 | 330,667 | 470,667 |
| 293,3 | 220,667 | 276 | 311,333 | 467,333 |
| 293,25 | 241,333 | 246,667 | 295,333 | 478,667 |
| 293,2 | 238 | 243,333 | 323,333 | 468 |
| 293,15 | 234,667 | 258 | 310,667 | 460,667 |
| 293,1 | 224,667 | 268,667 | 355,333 | 442,667 |
| 293,05 | 228,667 | 240 | 339,333 | 469,333 |
| 293 | 234 | 252,667 | 308,667 | 508,667 |
| 292,95 | 236 | 265,333 | 309,333 | 470 |
| 292,9 | 226,667 | 269,333 | 319,333 | 472,667 |
| 292,85 | 234 | 239,333 | 338 | 450,667 |
| 292,8 | 234,667 | 248 | 323,333 | 448,667 |
| 292,75 | 234 | 259,333 | 324 | 458 |
| 292,7 | 247,333 | 252 | 334 | 463,333 |
| 292,65 | 230,667 | 228,667 | 327,333 | 494,667 |
| 292,6 | 222,667 | 244 | 306 | 474 |
| 292,55 | 222,667 | 237,333 | 313,333 | 489,333 |
| 292,5 | 232 | 272,667 | 320 | 530 |
| 292,45 | 237,333 | 244,667 | 314,667 | 467,333 |
| 292,4 | 230,667 | 234,667 | 314 | 466 |
| 292,35 | 223,333 | 249,333 | 310,667 | 438 |
| 292,3 | 238 | 238 | 297,333 | 451,333 |
| 292,25 | 250,667 | 250,667 | 306 | 440 |
| 292,2 | 232 | 256,667 | 310,667 | 489,333 |
| 292,15 | 216,667 | 269,333 | 321,333 | 459,333 |
| 292,1 | 240,667 | 255,333 | 333,333 | 452 |
| 292,05 | 230,667 | 234 | 327,333 | 462 |
| 292 | 233,333 | 246 | 318 | 512 |
| 291,95 | 213,333 | 252 | 304,667 | 474 |
| 291,9 | 245,333 | 256 | 333,333 | 468,667 |
| 291,85 | 243,333 | 240 | 325,333 | 453,333 |
| 291,8 | 246 | 241,333 | 317,333 | 456,667 |
| 291,75 | 228,667 | 242,667 | 305,333 | 474,667 |
| 291,7 | 248,667 | 243,333 | 338 | 450 |
| 291,65 | 253,333 | 278 | 318 | 470,667 |
| 291,6 | 268,667 | 248,667 | 322 | 452 |
| 291,55 | 240,667 | 224,667 | 339,333 | 480 |
| 291,5 | 240,667 | 238 | 324 | 476,667 |
| 291,45 | 214 | 246 | 325,333 | 486,667 |
| 291,4 | 256 | 252,667 | 350,667 | 475,333 |
| 291,35 | 254 | 250,667 | 312 | 492,667 |
| 291,3 | 233,333 | 239,333 | 336,667 | 483,333 |
| 291,25 | 232,667 | 254 | 306 | 488 |
| 291,2 | 250,667 | 271,333 | 350,667 | 490 |
| 291,15 | 232,667 | 247,333 | 340 | 462 |
| 291,1 | 247,333 | 236,667 | 341,333 | 467,333 |
| 291,05 | 262 | 241,333 | 331,333 | 498,667 |
| 291 | 262 | 249,333 | 340,667 | 483,333 |
| 290,95 | 238 | 253,333 | 323,333 | 480,667 |
| 290,9 | 235,333 | 248 | 358 | 478 |
| 290,85 | 242,667 | 248,667 | 341,333 | 461,333 |
| 290,8 | 252,667 | 244,667 | 306,667 | 464,667 |
| 290,75 | 241,333 | 263,333 | 339,333 | 479,333 |
| 290,7 | 256 | 246,667 | 323,333 | 502,667 |
| 290,65 | 263,333 | 242,667 | 335,333 | 480,667 |
| 290,6 | 255,333 | 273,333 | 333,333 | 472 |
| 290,55 | 264,667 | 251,333 | 324,667 | 506,667 |
| 290,5 | 252 | 248,667 | 337,333 | 483,333 |
| 290,45 | 270 | 272 | 345,333 | 494,667 |
| 290,4 | 272,667 | 278 | 369,333 | 516 |
| 290,35 | 258,667 | 284,667 | 362 | 494 |
| 290,3 | 266 | 274 | 342,667 | 487,333 |
| 290,25 | 257,333 | 278,667 | 375,333 | 519,333 |
| 290,2 | 278,667 | 290 | 380 | 490,667 |
| 290,15 | 283,333 | 272 | 369,333 | 508 |
| 290,1 | 272 | 281,333 | 380 | 534,667 |
| 290,05 | 308,667 | 290 | 356 | 516 |
| 290 | 281,333 | 324,667 | 372,667 | 508,667 |
| 289,95 | 286 | 294 | 369,333 | 514 |
| 289,9 | 293,333 | 294 | 380 | 506,667 |
| 289,85 | 290 | 302,667 | 349,333 | 563,333 |
| 289,8 | 292,667 | 288 | 376 | 535,333 |
| 289,75 | 286,667 | 307,333 | 376,667 | 551,333 |
| 289,7 | 292 | 294 | 398 | 515,333 |
| 289,65 | 290,667 | 320 | 374 | 531,333 |
| 289,6 | 303,333 | 333,333 | 381,333 | 520 |
| 289,55 | 292 | 320,667 | 352,667 | 507,333 |
| 289,5 | 306 | 314,667 | 371,333 | 510,667 |
| 289,45 | 316,667 | 318 | 392 | 538,667 |
| 289,4 | 306 | 306 | 372,667 | 548,667 |
| 289,35 | 305,333 | 300 | 353,333 | 565,333 |
| 289,3 | 296 | 329,333 | 373,333 | 550,667 |
| 289,25 | 318 | 317,333 | 394 | 510,667 |
| 289,2 | 273,333 | 314 | 388 | 522 |
| 289,15 | 324 | 306 | 365,333 | 524 |
| 289,1 | 290 | 281,333 | 367,333 | 534 |
| 289,05 | 287,333 | 307,333 | 364 | 552,667 |
| 289 | 293,333 | 316,667 | 355,333 | 530,667 |
| 288,95 | 272,667 | 316 | 366,667 | 550,667 |
| 288,9 | 282 | 292,667 | 388,667 | 524 |
| 288,85 | 279,333 | 321,333 | 372 | 514,667 |
| 288,8 | 279,333 | 278,667 | 362,667 | 544,667 |
| 288,75 | 298 | 283,333 | 384 | 532,667 |
| 288,7 | 296,667 | 302,667 | 358 | 519,333 |
| 288,65 | 299,333 | 312 | 375,333 | 526,667 |
| 288,6 | 303,333 | 280 | 362,667 | 524 |
| 288,55 | 278 | 292 | 376,667 | 512 |
| 288,5 | 273,333 | 306 | 376,667 | 494 |
| 288,45 | 296,667 | 294 | 376,667 | 520 |
| 288,4 | 306 | 308 | 363,333 | 540 |
| 288,35 | 310 | 294 | 363,333 | 523,333 |
| 288,3 | 292 | 298,667 | 380 | 514 |
| 288,25 | 300 | 306 | 384,667 | 542 |
| 288,2 | 276,667 | 292 | 380 | 545,333 |
| 288,15 | 277,333 | 311,333 | 370,667 | 526 |
| 288,1 | 300 | 301,333 | 379,333 | 529,333 |
| 288,05 | 314 | 314,667 | 370 | 522 |
| 288 | 324,667 | 328 | 384 | 564,667 |
| 287,95 | 317,333 | 312 | 395,333 | 533,333 |
| 287,9 | 345,333 | 289,333 | 390 | 562 |
| 287,85 | 309,333 | 320 | 414,667 | 543,333 |
| 287,8 | 333,333 | 348 | 436 | 550 |
| 287,75 | 301,333 | 354,667 | 403,333 | 554,667 |
| 287,7 | 340,667 | 346,667 | 397,333 | 540 |
| 287,65 | 316,667 | 348,667 | 444,667 | 579,333 |
| 287,6 | 348 | 363,333 | 424 | 594,667 |
| 287,55 | 341,333 | 369,333 | 442,667 | 564,667 |
| 287,5 | 368 | 348,667 | 462,667 | 588 |
| 287,45 | 384 | 378,667 | 462 | 621,333 |
| 287,4 | 365,333 | 386 | 451,333 | 582,667 |
| 287,35 | 372,667 | 386,667 | 446 | 591,333 |
| 287,3 | 380,667 | 398 | 454 | 616,667 |
| 287,25 | 398 | 370,667 | 455,333 | 634 |
| 287,2 | 376 | 402,667 | 468,667 | 645,333 |
| 287,15 | 376 | 393,333 | 451,333 | 648,667 |
| 287,1 | 388 | 425,333 | 470 | 657,333 |
| 287,05 | 412 | 429,333 | 458,667 | 643,333 |
| 287 | 395,333 | 422,667 | 497,333 | 654,667 |
| 286,95 | 403,333 | 438,667 | 482 | 664,667 |
| 286,9 | 438 | 438 | 502,667 | 683,333 |
| 286,85 | 448 | 422 | 508,667 | 662,667 |
| 286,8 | 477,333 | 432 | 493,333 | 712 |
| 286,75 | 430 | 467,333 | 523,333 | 699,333 |
| 286,7 | 457,333 | 474 | 539,333 | 714 |
| 286,65 | 477,333 | 471,333 | 539,333 | 710,667 |
| 286,6 | 478 | 462 | 550 | 748 |
| 286,55 | 488 | 472 | 550 | 753,333 |
| 286,5 | 508 | 489,333 | 597,333 | 780,667 |
| 286,45 | 527,333 | 538,667 | 595,333 | 777,333 |
| 286,4 | 530 | 547,333 | 626 | 813,333 |
| 286,35 | 533,333 | 573,333 | 639,333 | 826,667 |
| 286,3 | 570,667 | 574,667 | 712 | 838 |
| 286,25 | 567,333 | 621,333 | 724,667 | 887,333 |
| 286,2 | 645,333 | 679,333 | 733,333 | 910,667 |
| 286,15 | 680,667 | 713,333 | 776 | 930 |
| 286,1 | 726,667 | 717,333 | 812 | 980,667 |
| 286,05 | 726 | 761,333 | 795,333 | 1024 |
| 286 | 760 | 810 | 872 | 1051,33 |
| 285,95 | 842,667 | 870,667 | 914,667 | 1090,67 |
| 285,9 | 856 | 914,667 | 967,333 | 1098 |
| 285,85 | 884 | 927,333 | 1015,33 | 1158,67 |
| 285,8 | 914,667 | 990 | 997,333 | 1150 |
| 285,75 | 948 | 1003,33 | 1105,33 | 1280,67 |
| 285,7 | 988 | 1022,67 | 1098 | 1302,67 |
| 285,65 | 1038 | 1065,33 | 1166,67 | 1302 |
| 285,6 | 1072,67 | 1109,33 | 1158 | 1383,33 |
| 285,55 | 1120 | 1080,67 | 1168,67 | 1383,33 |
| 285,5 | 1122 | 1126 | 1192,67 | 1375,33 |
| 285,45 | 1192,67 | 1166,67 | 1216 | 1401,33 |
| 285,4 | 1169,33 | 1146 | 1224 | 1428,67 |
| 285,35 | 1160 | 1152,67 | 1220,67 | 1418 |
| 285,3 | 1128 | 1144 | 1201,33 | 1403,33 |
| 285,25 | 1140 | 1122 | 1198 | 1400,67 |
| 285,2 | 1146,67 | 1100 | 1106,67 | 1354 |
| 285,15 | 1162,67 | 1100,67 | 1170 | 1318 |
| 285,1 | 1112 | 1038,67 | 1136,67 | 1335,33 |
| 285,05 | 1050 | 989,333 | 1085,33 | 1303,33 |
| 285 | 1092,67 | 965,333 | 1032,67 | 1254 |
| 284,95 | 1046 | 973,333 | 980,667 | 1198 |
| 284,9 | 968,667 | 923,333 | 968,667 | 1168 |
| 284,85 | 925,333 | 825,333 | 929,333 | 1148,67 |
| 284,8 | 901,333 | 821,333 | 837,333 | 1055,33 |
| 284,75 | 854 | 753,333 | 832,667 | 1003,33 |
| 284,7 | 792 | 714,667 | 832,667 | 965,333 |
| 284,65 | 724 | 672,667 | 765,333 | 952 |
| 284,6 | 686,667 | 667,333 | 736,667 | 906,667 |
| 284,55 | 616,667 | 595,333 | 675,333 | 887,333 |
| 284,5 | 600 | 553,333 | 619,333 | 838 |
| 284,45 | 546,667 | 512,667 | 596,667 | 754 |
| 284,4 | 533,333 | 513,333 | 573,333 | 773,333 |
| 284,35 | 485,333 | 469,333 | 532,667 | 706 |
| 284,3 | 458,667 | 448,667 | 514,667 | 714 |
| 284,25 | 437,333 | 414 | 500 | 676,667 |
| 284,2 | 378,667 | 374 | 484,667 | 629,333 |
| 284,15 | 370,667 | 374,667 | 477,333 | 599,333 |
| 284,1 | 320,667 | 350,667 | 420,667 | 582,667 |
| 284,05 | 322,667 | 313,333 | 376,667 | 574,667 |
| 284 | 306 | 322,667 | 364 | 530,667 |
| 283,95 | 292 | 304 | 363,333 | 536 |
| 283,9 | 270,667 | 302 | 381,333 | 537,333 |
| 283,85 | 264,667 | 287,333 | 354 | 506,667 |
| 283,8 | 259,333 | 273,333 | 321,333 | 486 |
| 283,75 | 240 | 268,667 | 343,333 | 545,333 |
| 283,7 | 241,333 | 281,333 | 343,333 | 504 |
| 283,65 | 237,333 | 254 | 368 | 486 |
| 283,6 | 212,667 | 252,667 | 339,333 | 518 |
| 283,55 | 234 | 233,333 | 310,667 | 493,333 |
| 283,5 | 223,333 | 247,333 | 342 | 460,667 |
| 283,45 | 188,667 | 241,333 | 302,667 | 464 |
| 283,4 | 208,667 | 246,667 | 299,333 | 464,667 |
| 283,35 | 198 | 234,667 | 306 | 466 |
| 283,3 | 198,667 | 226,667 | 294 | 452,667 |
| 283,25 | 195,333 | 229,333 | 294 | 493,333 |
| 283,2 | 200 | 230 | 288,667 | 473,333 |
| 283,15 | 198 | 211,333 | 296,667 | 446 |
| 283,1 | 201,333 | 222 | 303,333 | 479,333 |
| 283,05 | 216,667 | 238 | 290,667 | 482,667 |
| 283 | 194,667 | 224 | 270,667 | 456 |
| 282,95 | 202,667 | 232,667 | 286,667 | 457,333 |
| 282,9 | 186 | 222,667 | 305,333 | 431,333 |
| 282,85 | 195,333 | 210,667 | 284 | 437,333 |
| 282,8 | 200,667 | 216 | 297,333 | 478,667 |
| 282,75 | 211,333 | 216 | 306,667 | 450,667 |
| 282,7 | 177,333 | 226 | 287,333 | 432 |
| 282,65 | 190,667 | 207,333 | 278,667 | 447,333 |
| 282,6 | 200,667 | 214,667 | 286 | 450 |
| 282,55 | 190,667 | 208,667 | 308 | 442 |
| 282,5 | 212,667 | 219,333 | 287,333 | 445,333 |
| 282,45 | 190 | 218,667 | 286 | 443,333 |
| 282,4 | 203,333 | 223,333 | 290 | 414 |
| 282,35 | 194 | 222,667 | 281,333 | 422,667 |
| 282,3 | 191,333 | 225,333 | 296 | 428 |
| 282,25 | 188,667 | 218 | 272,667 | 412,667 |
| 282,2 | 198,667 | 209,333 | 324 | 412 |
| 282,15 | 194 | 214 | 294 | 418 |
| 282,1 | 194,667 | 230,667 | 301,333 | 430,667 |
| 282,05 | 181,333 | 208 | 292,667 | 453,333 |
| 282 | 172 | 197,333 | 300,667 | 444,667 |
| 281,95 | 204 | 204 | 291,333 | 450 |
| 281,9 | 208,667 | 228 | 292 | 406,667 |
| 281,85 | 198 | 203,333 | 276,667 | 435,333 |
| 281,8 | 180,667 | 208 | 287,333 | 391,333 |
| 281,75 | 192 | 218,667 | 277,333 | 419,333 |
| 281,7 | 196,667 | 202 | 286,667 | 403,333 |
| 281,65 | 182,667 | 214 | 288 | 445,333 |
| 281,6 | 190 | 222 | 266,667 | 434 |
| 281,55 | 213,333 | 202,667 | 275,333 | 413,333 |
| 281,5 | 203,333 | 225,333 | 271,333 | 430,667 |
| 281,45 | 188 | 223,333 | 272 | 424 |
| 281,4 | 164,667 | 198 | 281,333 | 417,333 |
| 281,35 | 181,333 | 208 | 272 | 400 |
| 281,3 | 182 | 218 | 306,667 | 402,667 |
| 281,25 | 183,333 | 225,333 | 278,667 | 403,333 |
| 281,2 | 184,667 | 223,333 | 296 | 428,667 |
| 281,15 | 194,667 | 220 | 274,667 | 427,333 |
| 281,1 | 183,333 | 211,333 | 276 | 424 |
| 281,05 | 192,667 | 208,667 | 275,333 | 447,333 |
| 281 | 206,667 | 202 | 292,667 | 427,333 |
| 280,95 | 196 | 212,667 | 288,667 | 398 |
| 280,9 | 174 | 213,333 | 258 | 430 |
| 280,85 | 176,667 | 230,667 | 288,667 | 416,667 |
| 280,8 | 178 | 222 | 273,333 | 418,667 |
| 280,75 | 180,667 | 209,333 | 310,667 | 401,333 |
| 280,7 | 190,667 | 212,667 | 298 | 428 |
| 280,65 | 198 | 204,667 | 288 | 431,333 |
| 280,6 | 186,667 | 208 | 274,667 | 429,333 |
| 280,55 | 183,333 | 232 | 272,667 | 416,667 |
| 280,5 | 187,333 | 221,333 | 296,667 | 416 |
| 280,45 | 186 | 213,333 | 281,333 | 428,667 |
| 280,4 | 202,667 | 190,667 | 280 | 435,333 |
| 280,35 | 190 | 202,667 | 274 | 413,333 |
| 280,3 | 182,667 | 202 | 279,333 | 388 |
| 280,25 | 192,667 | 202 | 285,333 | 402,667 |
| 280,2 | 192 | 206 | 268 | 409,333 |
| 280,15 | 185,333 | 222 | 294,667 | 400,667 |
| 280,1 | 201,333 | 235,333 | 297,333 | 395,333 |
| 280,05 | 180,667 | 217,333 | 264 | 425,333 |
| 280 | 186,667 | 201,333 | 293,333 | 427,333 |
| 279,95 | 176,667 | 222 | 286,667 | 416,667 |
| 279,9 | 192 | 210 | 286 | 431,333 |
| 279,85 | 188,667 | 219,333 | 272,667 | 441,333 |
| 279,8 | 196,667 | 208 | 267,333 | 436 |
| 279,75 | 195,333 | 212,667 | 308,667 | 428 |
| 279,7 | 201,333 | 214 | 294,667 | 451,333 |
| 279,65 | 202 | 200,667 | 262 | 385,333 |
| 279,6 | 188 | 232 | 286 | 425,333 |
| 279,55 | 166,667 | 220,667 | 294,667 | 386 |
| 279,5 | 172,667 | 195,333 | 290 | 420 |
| 279,45 | 187,333 | 212 | 284,667 | 406,667 |
| 279,4 | 190 | 205,333 | 282,667 | 416,667 |
| 279,35 | 191,333 | 200,667 | 279,333 | 420 |
| 279,3 | 189,333 | 210,667 | 289,333 | 381,333 |
| 279,25 | 188 | 209,333 | 295,333 | 399,333 |
| 279,2 | 182 | 202 | 274,667 | 416,667 |
| 279,15 | 199,333 | 214,667 | 271,333 | 425,333 |
| 279,1 | 184,667 | 210 | 261,333 | 406,667 |
| 279,05 | 188,667 | 200 | 263,333 | 452 |
| 279 | 174,667 | 186,667 | 268,667 | 464 |

## O1s

| Binding energy [eV] | TNTs | Se-Low | Se-Medium | Se-High |
| --- | --- | --- | --- | --- |
| 538 | 1358,86 | 1357,71 | 1546,57 | 1546,86 |
| 537,95 | 1330,29 | 1347,43 | 1544 | 1530,57 |
| 537,9 | 1328,29 | 1343,43 | 1550 | 1560,57 |
| 537,85 | 1313,71 | 1361,14 | 1548,57 | 1517,71 |
| 537,8 | 1320,57 | 1357,43 | 1550,29 | 1550 |
| 537,75 | 1296,57 | 1331,71 | 1570,57 | 1536,86 |
| 537,7 | 1319,14 | 1351,43 | 1545,71 | 1531,71 |
| 537,65 | 1322,29 | 1358 | 1488,57 | 1532,57 |
| 537,6 | 1330 | 1376 | 1555,43 | 1508,29 |
| 537,55 | 1338,29 | 1344 | 1549,14 | 1547,43 |
| 537,5 | 1312,29 | 1354,57 | 1565,14 | 1555,43 |
| 537,45 | 1321,71 | 1342,86 | 1538,57 | 1527,71 |
| 537,4 | 1327,71 | 1358,86 | 1567,71 | 1537,43 |
| 537,35 | 1326,29 | 1346 | 1512,57 | 1562,57 |
| 537,3 | 1333,71 | 1357,14 | 1526,29 | 1545,43 |
| 537,25 | 1335,71 | 1351,43 | 1557,43 | 1558 |
| 537,2 | 1332 | 1356,29 | 1523,71 | 1537,71 |
| 537,15 | 1325,14 | 1380,29 | 1540 | 1543,14 |
| 537,1 | 1322,86 | 1352,57 | 1556 | 1539,43 |
| 537,05 | 1359,71 | 1352 | 1551,14 | 1535,43 |
| 537 | 1338,29 | 1351,14 | 1568,57 | 1530,86 |
| 536,95 | 1358,86 | 1338 | 1556,86 | 1548,57 |
| 536,9 | 1323,43 | 1348 | 1552 | 1550,57 |
| 536,85 | 1334,86 | 1358,57 | 1542,57 | 1551,14 |
| 536,8 | 1343,71 | 1363,14 | 1566,57 | 1548,86 |
| 536,75 | 1345,71 | 1350 | 1577,71 | 1559,71 |
| 536,7 | 1365,43 | 1351,43 | 1568,57 | 1529,14 |
| 536,65 | 1370,86 | 1359,71 | 1540,86 | 1550,57 |
| 536,6 | 1310,86 | 1341,14 | 1594,29 | 1541,71 |
| 536,55 | 1328,86 | 1363,43 | 1551,14 | 1545,43 |
| 536,5 | 1347,14 | 1394,57 | 1564,86 | 1542,86 |
| 536,45 | 1329,71 | 1371,14 | 1564,86 | 1546,86 |
| 536,4 | 1321,71 | 1369,43 | 1545,71 | 1578,57 |
| 536,35 | 1283,43 | 1377,14 | 1561,14 | 1548,86 |
| 536,3 | 1312,57 | 1377,14 | 1564,86 | 1562,29 |
| 536,25 | 1327,43 | 1372 | 1558,57 | 1578,86 |
| 536,2 | 1334 | 1347,43 | 1591,14 | 1566,86 |
| 536,15 | 1306,57 | 1333,71 | 1563,71 | 1554,29 |
| 536,1 | 1330,57 | 1350,29 | 1577,43 | 1556,57 |
| 536,05 | 1317,71 | 1356 | 1558 | 1554,29 |
| 536 | 1324 | 1362 | 1534,57 | 1552,57 |
| 535,95 | 1320,57 | 1358,86 | 1540,29 | 1548,29 |
| 535,9 | 1313,14 | 1357,71 | 1518,86 | 1552 |
| 535,85 | 1297,43 | 1334 | 1521,71 | 1563,71 |
| 535,8 | 1294 | 1363,14 | 1554,57 | 1528 |
| 535,75 | 1297,43 | 1353,14 | 1548,57 | 1518,29 |
| 535,7 | 1312,57 | 1379,14 | 1563,43 | 1511,43 |
| 535,65 | 1330,29 | 1344,57 | 1558,29 | 1575,14 |
| 535,6 | 1327,43 | 1354,86 | 1584 | 1529,71 |
| 535,55 | 1308,86 | 1358,86 | 1560,29 | 1542 |
| 535,5 | 1294 | 1353,43 | 1583,43 | 1505,43 |
| 535,45 | 1316,86 | 1371,14 | 1538,29 | 1510 |
| 535,4 | 1314 | 1325,14 | 1543,43 | 1519,14 |
| 535,35 | 1316 | 1360,86 | 1564,86 | 1519,71 |
| 535,3 | 1303,71 | 1347,71 | 1540,29 | 1545,43 |
| 535,25 | 1306,29 | 1319,43 | 1539,71 | 1557,43 |
| 535,2 | 1336 | 1358,57 | 1534,29 | 1581,43 |
| 535,15 | 1317,14 | 1380,86 | 1582,57 | 1541,14 |
| 535,1 | 1295,71 | 1349,43 | 1555,71 | 1539,43 |
| 535,05 | 1301,14 | 1359,43 | 1574 | 1550,57 |
| 535 | 1299,71 | 1366,86 | 1553,71 | 1522 |
| 534,95 | 1321,43 | 1371,43 | 1568,86 | 1546 |
| 534,9 | 1336,57 | 1372,57 | 1571,14 | 1558 |
| 534,85 | 1322 | 1357,14 | 1548,29 | 1565,14 |
| 534,8 | 1336 | 1386 | 1569,14 | 1591,43 |
| 534,75 | 1335,71 | 1434,57 | 1610,86 | 1578,86 |
| 534,7 | 1328,86 | 1392,57 | 1602,57 | 1562,57 |
| 534,65 | 1319,71 | 1395,43 | 1620,57 | 1598,86 |
| 534,6 | 1329,71 | 1391,14 | 1616,57 | 1596,86 |
| 534,55 | 1360,57 | 1430,57 | 1624,29 | 1607,43 |
| 534,5 | 1386,86 | 1423,14 | 1624,29 | 1622 |
| 534,45 | 1399,14 | 1470,57 | 1640,86 | 1658,86 |
| 534,4 | 1400,86 | 1464 | 1643,14 | 1639,43 |
| 534,35 | 1405,14 | 1451,43 | 1685,43 | 1634 |
| 534,3 | 1434,57 | 1506,29 | 1720,57 | 1656,86 |
| 534,25 | 1455,43 | 1502,57 | 1715,71 | 1664,57 |
| 534,2 | 1465,14 | 1537,43 | 1707,14 | 1702 |
| 534,15 | 1480,29 | 1550 | 1776,57 | 1759,71 |
| 534,1 | 1514,86 | 1548,29 | 1794,57 | 1767,43 |
| 534,05 | 1566 | 1570,29 | 1830,57 | 1765,14 |
| 534 | 1541,14 | 1594,57 | 1853,14 | 1764,29 |
| 533,95 | 1548,86 | 1666,29 | 1885,71 | 1827,43 |
| 533,9 | 1579,71 | 1666 | 1902,29 | 1845,71 |
| 533,85 | 1595,71 | 1694,86 | 1929,71 | 1851,14 |
| 533,8 | 1591,71 | 1711,14 | 1976 | 1910 |
| 533,75 | 1637,71 | 1747,43 | 2009,71 | 1937,43 |
| 533,7 | 1651,43 | 1798,86 | 2032 | 1946,86 |
| 533,65 | 1679,14 | 1820,86 | 2093,14 | 2003,71 |
| 533,6 | 1754,29 | 1881,43 | 2152,86 | 2059,43 |
| 533,55 | 1760,86 | 1903,71 | 2213,71 | 2103,71 |
| 533,5 | 1811,14 | 2015,71 | 2234 | 2155,14 |
| 533,45 | 1890,29 | 2028,29 | 2325,43 | 2194,86 |
| 533,4 | 1938 | 2082,57 | 2335,14 | 2238,57 |
| 533,35 | 1915,43 | 2127,43 | 2427,71 | 2315,71 |
| 533,3 | 1977,71 | 2183,71 | 2482,29 | 2340,57 |
| 533,25 | 2011,43 | 2237,43 | 2590,29 | 2397,43 |
| 533,2 | 2065,43 | 2311,43 | 2570 | 2469,71 |
| 533,15 | 2107,43 | 2336 | 2631,71 | 2474,29 |
| 533,1 | 2121,71 | 2405,71 | 2711,71 | 2527,71 |
| 533,05 | 2123,43 | 2442,29 | 2730,29 | 2602,29 |
| 533 | 2184,86 | 2546 | 2800,57 | 2650 |
| 532,95 | 2240,57 | 2504,86 | 2877,43 | 2704 |
| 532,9 | 2272,29 | 2560,29 | 2942 | 2783,14 |
| 532,85 | 2302 | 2609,43 | 2947,71 | 2763,14 |
| 532,8 | 2369,43 | 2654,86 | 2950,29 | 2811,71 |
| 532,75 | 2397,71 | 2651,14 | 3037,14 | 2834 |
| 532,7 | 2416,86 | 2708,57 | 3053,71 | 2869,14 |
| 532,65 | 2449,14 | 2737,71 | 3106,86 | 2905,43 |
| 532,6 | 2478,86 | 2755,14 | 3090,29 | 2922,57 |
| 532,55 | 2474,57 | 2783,14 | 3137,14 | 2945,43 |
| 532,5 | 2494 | 2797,14 | 3179,14 | 2978,57 |
| 532,45 | 2532 | 2810,29 | 3157,71 | 2982,29 |
| 532,4 | 2558 | 2809,71 | 3176,29 | 2994,57 |
| 532,35 | 2548 | 2865,14 | 3162,57 | 2962,86 |
| 532,3 | 2607,71 | 2889,43 | 3140,57 | 2990,57 |
| 532,25 | 2613,14 | 2871,14 | 3165,71 | 3014 |
| 532,2 | 2636 | 2882,57 | 3185,43 | 3028,86 |
| 532,15 | 2603,43 | 2899,71 | 3226,57 | 3124,57 |
| 532,1 | 2654,86 | 2887,14 | 3230 | 3082,29 |
| 532,05 | 2720,29 | 2888,29 | 3217,71 | 3107,14 |
| 532 | 2718 | 2943,71 | 3258,86 | 3089,43 |
| 531,95 | 2795,14 | 3035,43 | 3289,14 | 3125,71 |
| 531,9 | 2834,86 | 3066,29 | 3346,86 | 3155,43 |
| 531,85 | 2906,86 | 3084,86 | 3449,43 | 3221,14 |
| 531,8 | 2985,43 | 3113,71 | 3514,29 | 3274,86 |
| 531,75 | 3098,86 | 3187,43 | 3598,29 | 3386,57 |
| 531,7 | 3228,29 | 3269,14 | 3716,86 | 3454,29 |
| 531,65 | 3295,43 | 3420,57 | 3781,43 | 3488,86 |
| 531,6 | 3398,29 | 3507,71 | 3987,14 | 3653,71 |
| 531,55 | 3670,86 | 3675,71 | 4141,43 | 3784 |
| 531,5 | 3769,71 | 3808 | 4338 | 3923,14 |
| 531,45 | 3938,86 | 3958,57 | 4490,86 | 4076 |
| 531,4 | 4138 | 4228 | 4752,29 | 4224,57 |
| 531,35 | 4348,57 | 4398,57 | 4927,71 | 4450 |
| 531,3 | 4637,71 | 4608 | 5207,71 | 4700 |
| 531,25 | 4891,43 | 4823,43 | 5442 | 4949,71 |
| 531,2 | 5080,57 | 5048,86 | 5689,14 | 5237,43 |
| 531,15 | 5328 | 5314,29 | 6026,57 | 5419,71 |
| 531,1 | 5618,57 | 5573,43 | 6307,14 | 5658,86 |
| 531,05 | 5865,14 | 5804 | 6523,71 | 5917,43 |
| 531 | 6048 | 6012 | 6815,71 | 6121,71 |
| 530,95 | 6269,71 | 6276,86 | 7050,57 | 6395,43 |
| 530,9 | 6402,29 | 6406,29 | 7254,57 | 6648,57 |
| 530,85 | 6563,71 | 6522,29 | 7432,57 | 6713,14 |
| 530,8 | 6677,14 | 6671,71 | 7495,71 | 6834,86 |
| 530,75 | 6707,14 | 6740,57 | 7556,29 | 6941,43 |
| 530,7 | 6721,43 | 6710,29 | 7552,86 | 6975,14 |
| 530,65 | 6664,57 | 6731,14 | 7491,71 | 6973,14 |
| 530,6 | 6579,14 | 6654,29 | 7487,71 | 6831,71 |
| 530,55 | 6486,57 | 6401,14 | 7286,86 | 6692 |
| 530,5 | 6234,86 | 6252,29 | 7053,71 | 6588,29 |
| 530,45 | 6000,86 | 5963,43 | 6751,14 | 6407,14 |
| 530,4 | 5677,71 | 5706 | 6446,57 | 6027,43 |
| 530,35 | 5310,29 | 5410,29 | 5988,29 | 5725,14 |
| 530,3 | 4980,86 | 5085,14 | 5716,86 | 5432 |
| 530,25 | 4646,29 | 4727,71 | 5327,14 | 5100,29 |
| 530,2 | 4225,43 | 4335,71 | 4938 | 4742,29 |
| 530,15 | 3901,43 | 4039,71 | 4483,71 | 4347,43 |
| 530,1 | 3565,43 | 3650,29 | 4095,14 | 4004,86 |
| 530,05 | 3217,43 | 3366 | 3729,14 | 3639,14 |
| 530 | 2946,29 | 3048,86 | 3439,71 | 3324,29 |
| 529,95 | 2666,29 | 2722,29 | 3125,14 | 3076,86 |
| 529,9 | 2419,71 | 2518,29 | 2829,43 | 2847,71 |
| 529,85 | 2210,86 | 2279,71 | 2576,29 | 2620 |
| 529,8 | 2032 | 2094,29 | 2347,14 | 2406 |
| 529,75 | 1877,14 | 1906,86 | 2210,57 | 2219,43 |
| 529,7 | 1741,43 | 1784 | 2065,43 | 2084,57 |
| 529,65 | 1640,57 | 1706,57 | 1902,86 | 1928,29 |
| 529,6 | 1575,71 | 1621,43 | 1815,14 | 1797,71 |
| 529,55 | 1473,71 | 1511,43 | 1706,86 | 1739,43 |
| 529,5 | 1390,57 | 1423,14 | 1604,86 | 1657,43 |
| 529,45 | 1322,29 | 1400,86 | 1568,29 | 1582,29 |
| 529,4 | 1313,71 | 1370,29 | 1526,86 | 1560 |
| 529,35 | 1267,14 | 1330 | 1468,57 | 1561,43 |
| 529,3 | 1236 | 1270 | 1459,43 | 1486,86 |
| 529,25 | 1220,86 | 1268,57 | 1448,86 | 1458 |
| 529,2 | 1222,29 | 1265,71 | 1431,43 | 1460,29 |
| 529,15 | 1214,57 | 1256,86 | 1402 | 1409,71 |
| 529,1 | 1191,71 | 1232,29 | 1362,29 | 1447,71 |
| 529,05 | 1168,86 | 1222,86 | 1388,57 | 1400,57 |
| 529 | 1153,71 | 1206,29 | 1364 | 1407,14 |
| 528,95 | 1159,71 | 1181,71 | 1352,29 | 1386,86 |
| 528,9 | 1144,57 | 1167,71 | 1306,29 | 1388,57 |
| 528,85 | 1170,86 | 1167,71 | 1346,57 | 1373,43 |
| 528,8 | 1159,14 | 1159,14 | 1339,14 | 1379,71 |
| 528,75 | 1151,14 | 1166,29 | 1355,14 | 1346,29 |
| 528,7 | 1125,14 | 1169,14 | 1343,71 | 1340,29 |
| 528,65 | 1125,14 | 1156 | 1324,57 | 1331,43 |
| 528,6 | 1146,29 | 1162,29 | 1316,29 | 1347,43 |
| 528,55 | 1141,14 | 1145,71 | 1319,71 | 1346 |
| 528,5 | 1135,71 | 1148,57 | 1317,43 | 1322,86 |
| 528,45 | 1125,71 | 1155,43 | 1330,86 | 1361,43 |
| 528,4 | 1095,71 | 1170,86 | 1305,14 | 1355,14 |
| 528,35 | 1127,14 | 1137,71 | 1288,29 | 1343,43 |
| 528,3 | 1112,86 | 1145,43 | 1300,86 | 1286 |
| 528,25 | 1118,29 | 1139,43 | 1283,71 | 1312,29 |
| 528,2 | 1105,14 | 1120,29 | 1282 | 1327,14 |
| 528,15 | 1101,14 | 1121,71 | 1271,43 | 1345,43 |
| 528,1 | 1136,29 | 1144 | 1302,86 | 1323,43 |
| 528,05 | 1089,71 | 1155,71 | 1287,71 | 1306 |
| 528 | 1094 | 1145,71 | 1254,86 | 1322,86 |
| 527,95 | 1103,71 | 1100,29 | 1274,29 | 1286,29 |
| 527,9 | 1111,71 | 1096,86 | 1273,14 | 1307,14 |
| 527,85 | 1084,57 | 1108 | 1253,14 | 1326,57 |
| 527,8 | 1070,57 | 1097,71 | 1235,43 | 1292 |
| 527,75 | 1091,43 | 1110,29 | 1252,86 | 1308,86 |
| 527,7 | 1069,43 | 1126,29 | 1245,71 | 1302,57 |
| 527,65 | 1102 | 1098,29 | 1273,71 | 1307,14 |
| 527,6 | 1073,14 | 1085,14 | 1259,71 | 1302 |
| 527,55 | 1085,14 | 1116,86 | 1265,14 | 1271,14 |
| 527,5 | 1089,14 | 1098 | 1255,71 | 1297,43 |
| 527,45 | 1051,43 | 1101,43 | 1230,86 | 1276,29 |
| 527,4 | 1078,86 | 1086,29 | 1242,86 | 1292,86 |
| 527,35 | 1098 | 1084,29 | 1250 | 1261,71 |
| 527,3 | 1094,29 | 1086 | 1266,86 | 1298,57 |
| 527,25 | 1058,86 | 1080,29 | 1275,43 | 1272,86 |
| 527,2 | 1065,71 | 1091,71 | 1263,71 | 1264,57 |
| 527,15 | 1072,86 | 1086,86 | 1266,57 | 1314,29 |
| 527,1 | 1079,71 | 1116,57 | 1256,57 | 1279,71 |
| 527,05 | 1086,29 | 1105,43 | 1247,71 | 1314,29 |
| 527 | 1075,43 | 1083,43 | 1262,86 | 1268 |
| 526,95 | 1078,86 | 1093,43 | 1256,86 | 1294,29 |
| 526,9 | 1064,57 | 1082,86 | 1253,43 | 1305,71 |
| 526,85 | 1060 | 1114,29 | 1237,14 | 1301,14 |
| 526,8 | 1062 | 1103,43 | 1252,29 | 1305,71 |
| 526,75 | 1074,29 | 1114,29 | 1246 | 1312,29 |
| 526,7 | 1074,29 | 1114 | 1273,14 | 1292,29 |
| 526,65 | 1065,43 | 1107,43 | 1254,57 | 1270,57 |
| 526,6 | 1063,14 | 1090,29 | 1245,43 | 1302,86 |
| 526,55 | 1071,14 | 1057,14 | 1260 | 1302,29 |
| 526,5 | 1050,57 | 1063,14 | 1249,14 | 1282,57 |
| 526,45 | 1089,14 | 1086,57 | 1242,57 | 1308,86 |
| 526,4 | 1073,14 | 1102,29 | 1220,57 | 1287,71 |
| 526,35 | 1083,14 | 1098 | 1225,14 | 1282 |
| 526,3 | 1046,86 | 1086,86 | 1234 | 1287,43 |
| 526,25 | 1074,57 | 1093,71 | 1235,71 | 1269,14 |
| 526,2 | 1066,57 | 1092,57 | 1228,86 | 1254,86 |
| 526,15 | 1061,14 | 1076 | 1225,71 | 1258 |
| 526,1 | 1050,86 | 1092,86 | 1234,57 | 1269,71 |
| 526,05 | 1046,29 | 1084,29 | 1259,14 | 1269,71 |
| 526 | 1035,71 | 1063,14 | 1226,86 | 1272 |
| 525,95 | 1047,71 | 1078,29 | 1242,29 | 1279,71 |
| 525,9 | 1082 | 1083,14 | 1247,43 | 1283,71 |
| 525,85 | 1056 | 1080,29 | 1224,29 | 1286 |
| 525,8 | 1055,71 | 1087,43 | 1222,29 | 1272,86 |
| 525,75 | 1074,57 | 1070,29 | 1225,43 | 1265,71 |
| 525,7 | 1067,14 | 1066,57 | 1226,86 | 1287,43 |
| 525,65 | 1084 | 1066,57 | 1237,14 | 1287,14 |
| 525,6 | 1062,57 | 1082 | 1253,43 | 1310,29 |
| 525,55 | 1060,86 | 1078,86 | 1243,43 | 1267,43 |
| 525,5 | 1064 | 1086,57 | 1233,43 | 1279,14 |
| 525,45 | 1069,43 | 1106,57 | 1225,14 | 1271,71 |
| 525,4 | 1070,57 | 1099,14 | 1263,43 | 1264,86 |
| 525,35 | 1079,14 | 1085,71 | 1256,86 | 1284,57 |
| 525,3 | 1054,57 | 1092 | 1245,14 | 1285,71 |
| 525,25 | 1056,29 | 1074,57 | 1213,43 | 1268,57 |
| 525,2 | 1085,14 | 1080,57 | 1199,71 | 1260,57 |
| 525,15 | 1066,86 | 1081,43 | 1215,14 | 1282 |
| 525,1 | 1036,57 | 1080,29 | 1236,29 | 1280,57 |
| 525,05 | 1051,71 | 1069,14 | 1220,29 | 1272,86 |
| 525 | 1064,57 | 1085,71 | 1249,71 | 1268,57 |
| 524,95 | 1053,43 | 1095,43 | 1249,71 | 1277,14 |
| 524,9 | 1049,14 | 1083,14 | 1269,14 | 1314 |
| 524,85 | 1054,29 | 1096,86 | 1240 | 1268,29 |
| 524,8 | 1063,14 | 1082,57 | 1260,29 | 1261,71 |
| 524,75 | 1066,57 | 1062,57 | 1241,71 | 1298,29 |
| 524,7 | 1046,57 | 1085,14 | 1238,57 | 1262 |
| 524,65 | 1080,29 | 1106,57 | 1228,86 | 1261,43 |
| 524,6 | 1071,71 | 1096 | 1249,71 | 1288,29 |
| 524,55 | 1072,29 | 1088,86 | 1238,86 | 1270,29 |
| 524,5 | 1069,14 | 1106 | 1235,71 | 1275,43 |
| 524,45 | 1051,43 | 1104,29 | 1240,57 | 1299,43 |
| 524,4 | 1044,29 | 1108,57 | 1229,71 | 1302,29 |
| 524,35 | 1086,86 | 1104,57 | 1263,71 | 1294,29 |
| 524,3 | 1080,86 | 1080,86 | 1236,29 | 1250,29 |
| 524,25 | 1043,14 | 1087,14 | 1270 | 1268 |
| 524,2 | 1048,57 | 1071,14 | 1212,57 | 1286 |
| 524,15 | 1090,29 | 1102,86 | 1226 | 1314,86 |
| 524,1 | 1063,71 | 1094,29 | 1244,57 | 1287,71 |
| 524,05 | 1077,14 | 1072,29 | 1260,29 | 1283,43 |
| 524 | 1045,14 | 1062,86 | 1265,71 | 1301,14 |
| 523,95 | 1068,57 | 1064 | 1236,29 | 1283,71 |
| 523,9 | 1101,14 | 1078,86 | 1254 | 1267,14 |
| 523,85 | 1046,29 | 1090 | 1222 | 1291,14 |
| 523,8 | 1066,86 | 1089,43 | 1265,71 | 1303,43 |
| 523,75 | 1052,57 | 1112 | 1234 | 1306,57 |
| 523,7 | 1060,29 | 1118 | 1240 | 1268,29 |
| 523,65 | 1057,71 | 1089,14 | 1237,14 | 1285,14 |
| 523,6 | 1076,86 | 1094,57 | 1223,71 | 1308 |
| 523,55 | 1068,57 | 1101,14 | 1254,57 | 1291,71 |
| 523,5 | 1079,43 | 1058,57 | 1239,14 | 1282,29 |
| 523,45 | 1068 | 1064,29 | 1217,71 | 1262 |
| 523,4 | 1061,71 | 1087,14 | 1243,43 | 1270,29 |
| 523,35 | 1076 | 1105,14 | 1261,71 | 1312,29 |
| 523,3 | 1077,43 | 1062,86 | 1208,57 | 1281,71 |
| 523,25 | 1053,14 | 1079,43 | 1233,43 | 1293,71 |
| 523,2 | 1064,29 | 1077,71 | 1226,29 | 1258,29 |
| 523,15 | 1058 | 1097,43 | 1223,14 | 1276,57 |
| 523,1 | 1071,43 | 1072,86 | 1214,86 | 1286 |
| 523,05 | 1065,14 | 1076,86 | 1223,43 | 1295,14 |
| 523 | 1082 | 1077,71 | 1229,43 | 1289,43 |

## Se3d

| Binding energy [eV] | TNTs | Se-Low | Se-Medium | Se-High |
| --- | --- | --- | --- | --- |
| 70 | 200,857 | 204 | 214,143 | 220,714 |
| 69,95 | 195,143 | 200,429 | 220,429 | 223,429 |
| 69,9 | 202,143 | 199,571 | 225,429 | 229,143 |
| 69,85 | 191,143 | 204,857 | 217,286 | 217,143 |
| 69,8 | 190,429 | 206,714 | 209,571 | 218 |
| 69,75 | 198 | 200,143 | 212,429 | 217,429 |
| 69,7 | 199 | 195,571 | 222,714 | 219,143 |
| 69,65 | 200,143 | 193,714 | 222,429 | 216 |
| 69,6 | 197,286 | 203,429 | 228,429 | 215,429 |
| 69,55 | 210,714 | 193,286 | 215 | 217,714 |
| 69,5 | 202,571 | 197,143 | 215 | 215 |
| 69,45 | 198,714 | 204,429 | 215,857 | 219 |
| 69,4 | 200,286 | 198,143 | 215,143 | 217,429 |
| 69,35 | 209,857 | 196,429 | 223,857 | 222,714 |
| 69,3 | 208 | 201,857 | 219,143 | 217,857 |
| 69,25 | 204,571 | 206,286 | 219,714 | 221,429 |
| 69,2 | 201,286 | 191,286 | 223,714 | 218 |
| 69,15 | 197,571 | 199 | 220,286 | 216,714 |
| 69,1 | 205,429 | 204,714 | 218,714 | 223,571 |
| 69,05 | 203,571 | 206,857 | 219,143 | 219 |
| 69 | 196,857 | 211,571 | 223 | 221 |
| 68,95 | 198,429 | 200,571 | 219,143 | 212,571 |
| 68,9 | 206,429 | 208 | 221 | 220,571 |
| 68,85 | 206,571 | 208,286 | 227,571 | 216 |
| 68,8 | 205,286 | 213,429 | 225,857 | 205,857 |
| 68,75 | 200,571 | 201,857 | 220,571 | 215,143 |
| 68,7 | 205,857 | 197,857 | 217,714 | 216,429 |
| 68,65 | 207,143 | 207 | 212,714 | 214 |
| 68,6 | 206 | 209,857 | 221,571 | 218 |
| 68,55 | 213,429 | 203,857 | 223,143 | 221,286 |
| 68,5 | 203,857 | 202,857 | 215,429 | 224,143 |
| 68,45 | 197,857 | 196,857 | 214,143 | 226,571 |
| 68,4 | 202,286 | 201,286 | 222,143 | 217,857 |
| 68,35 | 194 | 208,286 | 221,857 | 218,429 |
| 68,3 | 203,429 | 206,286 | 225,714 | 210 |
| 68,25 | 207,143 | 204,571 | 220,286 | 217,714 |
| 68,2 | 204 | 202,429 | 213,143 | 217,143 |
| 68,15 | 196,286 | 200,286 | 215,571 | 223,143 |
| 68,1 | 201,571 | 217,143 | 221,571 | 225,714 |
| 68,05 | 207,143 | 195,286 | 228,714 | 220,571 |
| 68 | 206 | 205 | 226,429 | 222,571 |
| 67,95 | 214,429 | 213,429 | 218,143 | 222,429 |
| 67,9 | 214,429 | 210 | 224,571 | 215,571 |
| 67,85 | 210,714 | 205,571 | 227,571 | 221,571 |
| 67,8 | 213,571 | 208,714 | 227,571 | 223 |
| 67,75 | 203,429 | 210,143 | 219,571 | 225,857 |
| 67,7 | 200,714 | 203,286 | 226 | 221,429 |
| 67,65 | 201,143 | 207,571 | 224,429 | 231 |
| 67,6 | 200,429 | 207 | 220,286 | 213,714 |
| 67,55 | 204,143 | 205,714 | 228 | 226,714 |
| 67,5 | 207,714 | 209,429 | 220,143 | 215,571 |
| 67,45 | 209,571 | 203,286 | 225,857 | 231,143 |
| 67,4 | 190,571 | 201,571 | 232 | 223 |
| 67,35 | 202,857 | 213,429 | 226,714 | 216,571 |
| 67,3 | 210,571 | 206 | 231,571 | 221,714 |
| 67,25 | 209,714 | 206,143 | 228,857 | 222,714 |
| 67,2 | 209,286 | 215,429 | 226,571 | 225 |
| 67,15 | 207,143 | 209,714 | 218 | 224,286 |
| 67,1 | 213,143 | 209,857 | 228,714 | 228,429 |
| 67,05 | 217 | 204,286 | 226,857 | 224,429 |
| 67 | 204,429 | 214,429 | 230,143 | 220,286 |
| 66,95 | 209,857 | 214,286 | 229,571 | 217 |
| 66,9 | 213,429 | 211,429 | 228,714 | 224,286 |
| 66,85 | 212,857 | 205,714 | 229,429 | 229,714 |
| 66,8 | 211,286 | 207,286 | 223,429 | 221,286 |
| 66,75 | 215,714 | 206 | 220,857 | 218,571 |
| 66,7 | 212,143 | 209,143 | 229,714 | 229,857 |
| 66,65 | 212,286 | 214,571 | 238,857 | 224,429 |
| 66,6 | 211,857 | 212,714 | 228,571 | 223,571 |
| 66,55 | 216,857 | 212,143 | 233,714 | 225,286 |
| 66,5 | 212,143 | 209 | 240,286 | 229,429 |
| 66,45 | 213,286 | 213,429 | 238 | 228,143 |
| 66,4 | 220,857 | 215,143 | 228,429 | 230,143 |
| 66,35 | 211,714 | 218,714 | 239,714 | 236,571 |
| 66,3 | 220,857 | 212,857 | 236,429 | 231 |
| 66,25 | 217,143 | 217,286 | 234 | 229,857 |
| 66,2 | 214,714 | 219,143 | 241,143 | 228,714 |
| 66,15 | 220 | 228,571 | 234,429 | 241,429 |
| 66,1 | 210,143 | 219,286 | 232,429 | 233,857 |
| 66,05 | 220,429 | 230,143 | 243 | 240 |
| 66 | 222,429 | 225,714 | 240,429 | 234,714 |
| 65,95 | 221,571 | 220,143 | 244,429 | 244,571 |
| 65,9 | 230,286 | 224,143 | 241,286 | 237,286 |
| 65,85 | 229,857 | 223,286 | 247,286 | 231,714 |
| 65,8 | 223,714 | 234,429 | 242,143 | 247,714 |
| 65,75 | 233,571 | 237,429 | 249,286 | 251,143 |
| 65,7 | 235,143 | 227,857 | 249,714 | 246,571 |
| 65,65 | 234,571 | 235,857 | 244,143 | 250,429 |
| 65,6 | 238 | 226,429 | 251,286 | 240,143 |
| 65,55 | 234,714 | 234,429 | 252,571 | 249,857 |
| 65,5 | 228 | 238,857 | 258,286 | 245,571 |
| 65,45 | 237 | 239,286 | 254,571 | 259,143 |
| 65,4 | 238 | 237,857 | 260 | 254,143 |
| 65,35 | 232,429 | 240,857 | 267,714 | 261,429 |
| 65,3 | 238,429 | 236,714 | 261,714 | 248 |
| 65,25 | 250,857 | 247,714 | 256,429 | 255 |
| 65,2 | 236 | 238,429 | 260,286 | 264 |
| 65,15 | 232,429 | 248,286 | 275,857 | 258,143 |
| 65,1 | 234,429 | 255,429 | 265 | 262,143 |
| 65,05 | 247,857 | 256,143 | 264,429 | 260,286 |
| 65 | 263,714 | 259,571 | 265,857 | 267,571 |
| 64,95 | 247,857 | 255,143 | 267 | 259,429 |
| 64,9 | 256,429 | 252,857 | 280,857 | 261,857 |
| 64,85 | 259 | 268 | 286,286 | 270,429 |
| 64,8 | 267,571 | 264 | 293,143 | 288,714 |
| 64,75 | 269,286 | 256,714 | 281,857 | 280,429 |
| 64,7 | 261,286 | 264,429 | 289,571 | 281,429 |
| 64,65 | 265,857 | 274 | 296,143 | 281,143 |
| 64,6 | 261 | 279,286 | 301,857 | 290,714 |
| 64,55 | 269,714 | 284,571 | 297 | 284,429 |
| 64,5 | 265,857 | 281,143 | 301,571 | 295 |
| 64,45 | 273,857 | 290,857 | 312,286 | 298 |
| 64,4 | 283 | 284,286 | 314,571 | 295,714 |
| 64,35 | 282 | 296,571 | 328,571 | 305,857 |
| 64,3 | 290,857 | 296,143 | 333 | 319,429 |
| 64,25 | 289,857 | 303,429 | 319,143 | 321 |
| 64,2 | 300,429 | 310,143 | 335,571 | 331,286 |
| 64,15 | 302,429 | 303,143 | 341,429 | 332,857 |
| 64,1 | 311,857 | 312,857 | 345,143 | 327,143 |
| 64,05 | 311,143 | 320,286 | 351 | 325,857 |
| 64 | 311,857 | 337 | 342,429 | 335,714 |
| 63,95 | 320,429 | 340,571 | 358,286 | 337 |
| 63,9 | 320,571 | 334,714 | 362,286 | 345,286 |
| 63,85 | 333,571 | 339,571 | 370,857 | 355,714 |
| 63,8 | 341,286 | 342,714 | 373,571 | 359,143 |
| 63,75 | 352,857 | 338 | 385,143 | 358,429 |
| 63,7 | 351,714 | 353,857 | 385,429 | 370,714 |
| 63,65 | 354,143 | 365,143 | 380,571 | 379 |
| 63,6 | 361,429 | 366,571 | 398,571 | 381,714 |
| 63,55 | 369,714 | 367 | 398,571 | 380,857 |
| 63,5 | 357,714 | 368,429 | 396,571 | 383,857 |
| 63,45 | 359,429 | 378,429 | 413,714 | 397,286 |
| 63,4 | 372,143 | 384 | 414,571 | 399,857 |
| 63,35 | 381 | 376 | 400,857 | 398,429 |
| 63,3 | 379,143 | 383,714 | 420,143 | 390,286 |
| 63,25 | 396,571 | 394,143 | 414,857 | 406,857 |
| 63,2 | 404,143 | 417,429 | 416,143 | 404,286 |
| 63,15 | 398,143 | 403 | 421,571 | 405 |
| 63,1 | 407,143 | 399,429 | 421,571 | 414,143 |
| 63,05 | 407 | 395,143 | 421,143 | 413 |
| 63 | 401,714 | 408,714 | 436,143 | 421,429 |
| 62,95 | 393 | 389,571 | 432,571 | 413,571 |
| 62,9 | 390,857 | 398,143 | 427,571 | 406,857 |
| 62,85 | 385,286 | 391,571 | 414,143 | 403,857 |
| 62,8 | 379,714 | 393,857 | 422,429 | 415,571 |
| 62,75 | 393,286 | 393,429 | 425,571 | 416,714 |
| 62,7 | 388,857 | 383,714 | 418,857 | 418,286 |
| 62,65 | 376,571 | 393,571 | 409 | 407,714 |
| 62,6 | 363,714 | 383,571 | 411,286 | 390,857 |
| 62,55 | 378,429 | 386 | 409,857 | 391,429 |
| 62,5 | 372,857 | 378,286 | 407,714 | 385 |
| 62,45 | 359 | 377,714 | 402,714 | 383,857 |
| 62,4 | 354,857 | 371,857 | 393,857 | 375,714 |
| 62,35 | 355,143 | 358,143 | 383,429 | 370 |
| 62,3 | 350,143 | 363,571 | 369,143 | 375,571 |
| 62,25 | 345,714 | 344,286 | 370 | 360,286 |
| 62,2 | 335 | 338,857 | 362,143 | 362,286 |
| 62,15 | 325,143 | 333,143 | 358,857 | 355,857 |
| 62,1 | 331,714 | 339,571 | 354,429 | 345 |
| 62,05 | 316,714 | 342,857 | 352,143 | 340,143 |
| 62 | 318,286 | 324,143 | 359 | 337,714 |
| 61,95 | 310,286 | 302,429 | 334 | 332,143 |
| 61,9 | 305 | 314,143 | 335,571 | 314,714 |
| 61,85 | 299,429 | 306,143 | 323,286 | 312,571 |
| 61,8 | 296,571 | 293,857 | 323 | 306,857 |
| 61,75 | 295,857 | 301,571 | 321 | 306,286 |
| 61,7 | 286,857 | 285,143 | 318,714 | 309 |
| 61,65 | 284,857 | 286 | 304 | 296,286 |
| 61,6 | 274,857 | 284 | 294,429 | 282,286 |
| 61,55 | 267,286 | 272,143 | 292 | 286,286 |
| 61,5 | 266 | 274,857 | 290,857 | 281,857 |
| 61,45 | 271,143 | 272,714 | 291,857 | 276,429 |
| 61,4 | 261,143 | 265,571 | 288,286 | 275,143 |
| 61,35 | 260,429 | 256,714 | 285 | 267,857 |
| 61,3 | 246,143 | 257,143 | 277,857 | 268,429 |
| 61,25 | 257,286 | 260,286 | 279,857 | 265 |
| 61,2 | 251,571 | 254,571 | 275,571 | 260,429 |
| 61,15 | 240,143 | 246,429 | 270 | 267,714 |
| 61,1 | 234,429 | 238,429 | 261 | 259 |
| 61,05 | 244,857 | 239,143 | 257,143 | 255,429 |
| 61 | 240,286 | 233,286 | 259,571 | 246,857 |
| 60,95 | 236,143 | 234,143 | 261,571 | 257,286 |
| 60,9 | 233 | 238,429 | 255,714 | 247,286 |
| 60,85 | 226,429 | 234,857 | 254,571 | 247 |
| 60,8 | 234,571 | 226,429 | 256,714 | 235,714 |
| 60,75 | 232 | 218,714 | 248 | 243 |
| 60,7 | 219,857 | 225,857 | 249,429 | 246,429 |
| 60,65 | 213,286 | 226,286 | 244,571 | 235,571 |
| 60,6 | 218,429 | 231,857 | 237,857 | 244 |
| 60,55 | 219,857 | 220,571 | 233,571 | 237,571 |
| 60,5 | 211,143 | 212,571 | 236,714 | 236,714 |
| 60,45 | 207,143 | 221,143 | 231,429 | 226,714 |
| 60,4 | 208,714 | 216,286 | 227,857 | 227,571 |
| 60,35 | 205,286 | 221,143 | 228,857 | 227,857 |
| 60,3 | 206,571 | 208,714 | 226,143 | 227,571 |
| 60,25 | 204,571 | 207,429 | 229,286 | 220,857 |
| 60,2 | 200,714 | 202,143 | 224,857 | 220,286 |
| 60,15 | 211,429 | 205,429 | 224,714 | 226,143 |
| 60,1 | 200,286 | 200,571 | 223,286 | 223,571 |
| 60,05 | 201,429 | 199 | 218,714 | 217,714 |
| 60 | 199,714 | 208 | 223,857 | 213,571 |
| 59,95 | 201 | 200,571 | 222 | 217,857 |
| 59,9 | 204,857 | 193,429 | 220,571 | 210,286 |
| 59,85 | 198,857 | 204,143 | 213,571 | 217 |
| 59,8 | 194,571 | 198,429 | 211,286 | 215,857 |
| 59,75 | 195 | 198,571 | 219,571 | 211,571 |
| 59,7 | 199,286 | 204 | 210,286 | 208,857 |
| 59,65 | 192,286 | 197,714 | 213,286 | 210,286 |
| 59,6 | 190,857 | 189,571 | 211,143 | 208,429 |
| 59,55 | 188,714 | 191,286 | 209,857 | 206 |
| 59,5 | 188,429 | 188,714 | 199,857 | 216 |
| 59,45 | 191,857 | 195 | 202,571 | 211,286 |
| 59,4 | 178 | 187,857 | 206,286 | 194,286 |
| 59,35 | 187,143 | 190,143 | 212,571 | 208,429 |
| 59,3 | 192,571 | 193,857 | 204,571 | 217,143 |
| 59,25 | 192,286 | 196,714 | 214,571 | 205,429 |
| 59,2 | 191,286 | 190,429 | 211,857 | 199,714 |
| 59,15 | 186,286 | 187 | 209 | 202,286 |
| 59,1 | 191 | 195,286 | 210,429 | 200,143 |
| 59,05 | 180,143 | 186,286 | 211,143 | 199,714 |
| 59 | 181,571 | 189,143 | 205,571 | 199,286 |
| 58,95 | 196,571 | 189,714 | 207,571 | 211 |
| 58,9 | 185,286 | 183 | 201,143 | 203,429 |
| 58,85 | 183,286 | 186,429 | 207,286 | 210,143 |
| 58,8 | 189 | 186,857 | 190,857 | 196 |
| 58,75 | 182,714 | 180,429 | 203,714 | 196,143 |
| 58,7 | 172,286 | 181,143 | 200 | 194,429 |
| 58,65 | 182 | 180,714 | 203,286 | 193,143 |
| 58,6 | 182 | 193,143 | 196,714 | 201,143 |
| 58,55 | 183,571 | 184 | 189,571 | 197,429 |
| 58,5 | 177,714 | 185,857 | 200,857 | 201,429 |
| 58,45 | 182,429 | 177,714 | 197,286 | 203,143 |
| 58,4 | 169,143 | 188,143 | 196,571 | 198,571 |
| 58,35 | 183,286 | 186,714 | 194,714 | 203,857 |
| 58,3 | 186,429 | 179,714 | 189,143 | 196,714 |
| 58,25 | 179 | 181,571 | 194,571 | 187,429 |
| 58,2 | 174,714 | 181 | 203,286 | 202,714 |
| 58,15 | 175 | 174 | 195,143 | 198,286 |
| 58,1 | 176,857 | 183,857 | 201,714 | 194,286 |
| 58,05 | 175,714 | 184 | 198,714 | 194,143 |
| 58 | 176,286 | 177,286 | 200,286 | 193 |
| 57,95 | 170,857 | 181,143 | 196,143 | 198,714 |
| 57,9 | 176 | 186 | 195 | 205,571 |
| 57,85 | 168,286 | 176,286 | 200,714 | 195,857 |
| 57,8 | 176,286 | 171,571 | 191,857 | 202,571 |
| 57,75 | 177,286 | 175,429 | 196,857 | 200,286 |
| 57,7 | 173,143 | 175,429 | 193 | 197,714 |
| 57,65 | 173,857 | 171 | 197,571 | 195,714 |
| 57,6 | 173,429 | 172,571 | 200,571 | 195 |
| 57,55 | 177,714 | 181,714 | 183,714 | 194,143 |
| 57,5 | 176,143 | 179,429 | 200,143 | 200,286 |
| 57,45 | 169,286 | 175,429 | 190,571 | 195,286 |
| 57,4 | 178,714 | 180,857 | 193,429 | 195,429 |
| 57,35 | 165,429 | 179,857 | 197 | 197,571 |
| 57,3 | 169,429 | 181,286 | 191,286 | 204,571 |
| 57,25 | 179,143 | 181,286 | 191,714 | 208,571 |
| 57,2 | 173,143 | 178,429 | 191,571 | 203,286 |
| 57,15 | 172,857 | 178,429 | 197 | 210,286 |
| 57,1 | 168,857 | 187,143 | 199,286 | 213,286 |
| 57,05 | 177,143 | 179,714 | 203 | 205 |
| 57 | 169,857 | 184,571 | 200,286 | 219,143 |
| 56,95 | 172 | 176,429 | 197,571 | 221,143 |
| 56,9 | 172,286 | 181,571 | 200 | 232,429 |
| 56,85 | 172 | 177,857 | 204,286 | 239,857 |
| 56,8 | 176,571 | 178,143 | 204,857 | 255 |
| 56,75 | 171,857 | 176,429 | 216,714 | 262,571 |
| 56,7 | 167 | 178 | 209,286 | 271,571 |
| 56,65 | 173,857 | 182,571 | 212,429 | 278,714 |
| 56,6 | 169,286 | 179,857 | 217,143 | 288 |
| 56,55 | 165,857 | 181,143 | 221,429 | 296,857 |
| 56,5 | 176,714 | 183,714 | 226 | 316,429 |
| 56,45 | 173 | 178,714 | 223 | 308,429 |
| 56,4 | 172,857 | 175,714 | 230,571 | 318,286 |
| 56,35 | 169,857 | 178,714 | 231,143 | 310,429 |
| 56,3 | 176,429 | 180,857 | 242,571 | 313,143 |
| 56,25 | 174,857 | 180,714 | 223,286 | 306 |
| 56,2 | 181 | 188,571 | 223,571 | 299,857 |
| 56,15 | 172,286 | 189,143 | 222,714 | 293,857 |
| 56,1 | 168,429 | 186,429 | 223,857 | 284,857 |
| 56,05 | 168,286 | 178,857 | 228 | 281,286 |
| 56 | 170,286 | 184,429 | 222,429 | 293 |
| 55,95 | 181,286 | 186,429 | 223,143 | 290,286 |
| 55,9 | 171,857 | 183,857 | 221,143 | 300,429 |
| 55,85 | 171 | 186,857 | 229 | 305,143 |
| 55,8 | 177,286 | 190,714 | 225,286 | 317,143 |
| 55,75 | 165,286 | 181,714 | 233,429 | 335,429 |
| 55,7 | 175,714 | 185 | 228,429 | 338,286 |
| 55,65 | 174,429 | 180,286 | 232 | 344,143 |
| 55,6 | 170,714 | 183,143 | 232,857 | 345 |
| 55,55 | 180,143 | 186,857 | 236,143 | 341,429 |
| 55,5 | 171,571 | 187,143 | 232,857 | 334,857 |
| 55,45 | 178,286 | 183,714 | 239,571 | 329 |
| 55,4 | 170,286 | 188,857 | 234,143 | 316,857 |
| 55,35 | 174,714 | 177,714 | 228,857 | 293 |
| 55,3 | 172,714 | 171,714 | 226,429 | 273,429 |
| 55,25 | 172,857 | 174,571 | 219,143 | 275,429 |
| 55,2 | 169,857 | 182,857 | 213,286 | 252,714 |
| 55,15 | 178 | 179,714 | 210,429 | 246,143 |
| 55,1 | 171 | 178,571 | 218 | 228,714 |
| 55,05 | 177,286 | 180 | 211,571 | 216,286 |
| 55 | 172 | 178,429 | 210,143 | 218,714 |
| 54,95 | 179,571 | 184,143 | 207,571 | 203,714 |
| 54,9 | 174 | 185,571 | 206,571 | 211,571 |
| 54,85 | 174 | 182,714 | 203,286 | 207 |
| 54,8 | 173 | 189 | 201,286 | 209 |
| 54,75 | 181,571 | 175,857 | 207,429 | 201,571 |
| 54,7 | 172,429 | 192,571 | 201,429 | 203,143 |
| 54,65 | 172 | 186,143 | 197,143 | 193,286 |
| 54,6 | 171,143 | 187,429 | 199,714 | 195,571 |
| 54,55 | 177,714 | 186 | 203,143 | 206,714 |
| 54,5 | 171,143 | 183,857 | 200,571 | 195,857 |
| 54,45 | 179,286 | 179,714 | 191,143 | 198 |
| 54,4 | 172,571 | 180,714 | 196 | 196,571 |
| 54,35 | 178,143 | 165 | 203,429 | 199,143 |
| 54,3 | 174,571 | 176,143 | 206,429 | 193,286 |
| 54,25 | 175,857 | 179,143 | 199,429 | 195 |
| 54,2 | 174,571 | 177,857 | 207,857 | 188,143 |
| 54,15 | 174,286 | 180,286 | 211,429 | 195,286 |
| 54,1 | 179,857 | 181,429 | 191,714 | 196,286 |
| 54,05 | 182 | 182,286 | 191,714 | 189,429 |
| 54 | 172 | 183,857 | 191 | 195,571 |
| 53,95 | 182,143 | 179,857 | 201,143 | 196,143 |
| 53,9 | 179,714 | 179,429 | 198,857 | 201,714 |
| 53,85 | 184,143 | 190,857 | 194 | 200 |
| 53,8 | 186,571 | 182,571 | 200,714 | 193 |
| 53,75 | 188,571 | 185,286 | 203,714 | 192,571 |
| 53,7 | 177,857 | 185,286 | 197,143 | 197,143 |
| 53,65 | 184,714 | 183,286 | 202,571 | 192,429 |
| 53,6 | 192,286 | 183,714 | 195,286 | 191,857 |
| 53,55 | 186,143 | 182,286 | 200,857 | 191,143 |
| 53,5 | 181,143 | 191,571 | 203,571 | 193,571 |
| 53,45 | 196 | 185,571 | 204,429 | 194,857 |
| 53,4 | 178,714 | 186,857 | 199 | 192,429 |
| 53,35 | 192,286 | 194,143 | 203,857 | 191 |
| 53,3 | 186,571 | 186,143 | 201,143 | 198,714 |
| 53,25 | 187,143 | 197 | 196,714 | 186,286 |
| 53,2 | 194 | 190 | 198,571 | 183,714 |
| 53,15 | 194,571 | 196 | 208,143 | 190 |
| 53,1 | 188,714 | 194,714 | 195,571 | 192,286 |
| 53,05 | 193,429 | 194,857 | 198,143 | 195,571 |
| 53 | 191 | 195,857 | 205,714 | 196 |
| 52,95 | 200,714 | 193,143 | 202,714 | 194,714 |
| 52,9 | 194,286 | 193,571 | 209,286 | 198,429 |
| 52,85 | 200,143 | 192,857 | 210,429 | 191,286 |
| 52,8 | 200,429 | 195,429 | 215,857 | 195,857 |
| 52,75 | 194,857 | 192 | 206,857 | 201,857 |
| 52,7 | 196,429 | 194,143 | 209,857 | 199 |
| 52,65 | 196,857 | 195,143 | 204,571 | 203,571 |
| 52,6 | 199,429 | 200,714 | 214,286 | 200,714 |
| 52,55 | 200,571 | 189,429 | 209 | 191,429 |
| 52,5 | 199,143 | 201 | 210,857 | 194,714 |
| 52,45 | 197 | 200,429 | 205,857 | 198,571 |
| 52,4 | 196,571 | 201,429 | 211,714 | 203 |
| 52,35 | 197,429 | 195,429 | 208 | 201,286 |
| 52,3 | 201,714 | 195,429 | 214 | 205,429 |
| 52,25 | 190,143 | 197,429 | 215,429 | 202,286 |
| 52,2 | 191,714 | 207,714 | 217,857 | 205,429 |
| 52,15 | 200,857 | 196,857 | 219,571 | 203,714 |
| 52,1 | 196,286 | 200,857 | 218,857 | 207,714 |
| 52,05 | 199,429 | 205 | 221,857 | 205,429 |
| 52 | 199,286 | 200,286 | 214,571 | 208,429 |
| 51,95 | 210,286 | 203,286 | 213,857 | 204,429 |
| 51,9 | 210,429 | 208,857 | 224,143 | 206,286 |
| 51,85 | 205,143 | 205,714 | 215,571 | 208,571 |
| 51,8 | 203,286 | 200 | 221,429 | 211,571 |
| 51,75 | 196,857 | 206,857 | 219,286 | 208 |
| 51,7 | 204,286 | 201,714 | 218,429 | 198,857 |
| 51,65 | 200,714 | 202,714 | 210,429 | 211,714 |
| 51,6 | 196,143 | 207,429 | 218,429 | 199 |
| 51,55 | 208,286 | 198,286 | 219,857 | 207,143 |
| 51,5 | 194,714 | 205,714 | 215 | 198,429 |
| 51,45 | 200,857 | 201,429 | 209,286 | 203,286 |
| 51,4 | 204,286 | 211,143 | 213,857 | 205,571 |
| 51,35 | 204,143 | 205,429 | 225,571 | 202,714 |
| 51,3 | 201,857 | 208,143 | 208,571 | 214,429 |
| 51,25 | 204 | 207,429 | 221,714 | 199,571 |
| 51,2 | 203,571 | 203,571 | 218,286 | 211,429 |
| 51,15 | 205,714 | 201,714 | 210,429 | 201,571 |
| 51,1 | 207,857 | 209,429 | 223 | 204,714 |
| 51,05 | 202,429 | 207,571 | 223,143 | 204,714 |
| 51 | 202,857 | 205,286 | 214,286 | 210,143 |
| 50,95 | 198,571 | 207,143 | 213,857 | 206 |
| 50,9 | 197 | 192,286 | 214,714 | 197,571 |
| 50,85 | 201,857 | 188,429 | 208,714 | 198 |
| 50,8 | 197,714 | 196,143 | 214,714 | 203 |
| 50,75 | 200,714 | 200,571 | 204,857 | 205,286 |
| 50,7 | 198,143 | 200,143 | 206,286 | 203,286 |
| 50,65 | 193 | 193,143 | 213,714 | 194,429 |
| 50,6 | 192,143 | 197,429 | 207,714 | 203,571 |
| 50,55 | 185,571 | 200 | 205 | 191 |
| 50,5 | 190,143 | 200,286 | 203,857 | 192,286 |
| 50,45 | 192,429 | 192,143 | 202,714 | 204,143 |
| 50,4 | 195,143 | 190,143 | 201,286 | 190,429 |
| 50,35 | 189 | 186 | 201,143 | 191,571 |
| 50,3 | 187,714 | 184,143 | 212,143 | 193,143 |
| 50,25 | 187,286 | 188,143 | 201,286 | 191,143 |
| 50,2 | 180,571 | 195,857 | 198,571 | 187,857 |
| 50,15 | 180,571 | 193 | 199,429 | 188 |
| 50,1 | 183,429 | 185,429 | 199,714 | 186,857 |
| 50,05 | 183 | 177 | 203,714 | 184,571 |
| 50 | 183,286 | 185,429 | 195,429 | 187,143 |
| 49,95 | 172,429 | 184,429 | 196,714 | 191,143 |
| 49,9 | 172,857 | 176,857 | 189,143 | 190,429 |
| 49,85 | 169 | 181 | 191,429 | 185,429 |
| 49,8 | 172,714 | 180 | 191,143 | 173,857 |
| 49,75 | 178 | 176,286 | 191,714 | 179,857 |
| 49,7 | 174,714 | 177,857 | 190,143 | 173 |
| 49,65 | 163 | 176,143 | 191,714 | 178,286 |
| 49,6 | 170,429 | 177,857 | 191,857 | 183,714 |
| 49,55 | 174,286 | 175,571 | 184,143 | 172,286 |
| 49,5 | 169,143 | 174,857 | 186 | 174,857 |
| 49,45 | 174,429 | 167 | 178,286 | 168 |
| 49,4 | 170,714 | 173,143 | 186,143 | 180 |
| 49,35 | 173 | 172,286 | 183,571 | 170,143 |
| 49,3 | 161 | 170,286 | 176,857 | 176,429 |
| 49,25 | 165,857 | 166,143 | 177,286 | 177 |
| 49,2 | 164,714 | 164,143 | 178,857 | 171,286 |
| 49,15 | 169,714 | 166,143 | 175,857 | 172 |
| 49,1 | 169 | 162 | 180,714 | 168,143 |
| 49,05 | 161,429 | 164,286 | 174,571 | 164,286 |
| 49 | 164,429 | 171,143 | 171,714 | 167,429 |
| 48,95 | 159,429 | 166,429 | 174,857 | 171,429 |
| 48,9 | 156,571 | 161 | 167,429 | 165,286 |
| 48,85 | 156 | 164,857 | 177,143 | 162 |
| 48,8 | 155,429 | 162,286 | 171,571 | 168 |
| 48,75 | 167,714 | 152 | 180,286 | 165,857 |
| 48,7 | 165,143 | 162,571 | 168,143 | 161,286 |
| 48,65 | 162,286 | 165,714 | 169,286 | 164,857 |
| 48,6 | 153,571 | 156,429 | 167,143 | 164,143 |
| 48,55 | 156 | 148,143 | 169,286 | 161 |
| 48,5 | 156,429 | 155 | 175 | 160,714 |
| 48,45 | 158,571 | 157,286 | 167,429 | 162,143 |
| 48,4 | 154,429 | 157,571 | 172,857 | 154,286 |
| 48,35 | 157,571 | 149,857 | 157,571 | 162 |
| 48,3 | 158,286 | 156,714 | 165,143 | 156,857 |
| 48,25 | 149,571 | 155,286 | 157 | 161,857 |
| 48,2 | 160,571 | 157,714 | 163 | 164,286 |
| 48,15 | 147,857 | 153,571 | 170,143 | 161 |
| 48,1 | 157,571 | 149,714 | 162,571 | 161,143 |
| 48,05 | 156,429 | 147,857 | 154,857 | 169,143 |
| 48 | 151,286 | 149,571 | 160,143 | 157,571 |
| 47,95 | 150,857 | 150 | 160,714 | 143,429 |
| 47,9 | 150,571 | 146,714 | 171,143 | 160 |
| 47,85 | 149,714 | 153,429 | 165 | 152,143 |
| 47,8 | 151,857 | 153,429 | 161,429 | 157,143 |
| 47,75 | 151,286 | 150 | 161,571 | 155 |
| 47,7 | 147,286 | 145,143 | 155 | 157 |
| 47,65 | 149,857 | 143,714 | 157,571 | 158,286 |
| 47,6 | 148,714 | 152,143 | 162,714 | 155,571 |
| 47,55 | 145,571 | 150,857 | 156,571 | 151,429 |
| 47,5 | 146,286 | 143,429 | 165,857 | 154,143 |
| 47,45 | 144,857 | 146,143 | 161,714 | 151 |
| 47,4 | 147,857 | 150,571 | 148,571 | 148,143 |
| 47,35 | 146 | 145,857 | 157,571 | 148,571 |
| 47,3 | 138 | 140,714 | 157,857 | 147,429 |
| 47,25 | 143,143 | 145,571 | 158,429 | 147,286 |
| 47,2 | 140,857 | 144,857 | 147,429 | 143,571 |
| 47,15 | 137,571 | 151 | 154,571 | 146,857 |
| 47,1 | 142,429 | 149,429 | 149,571 | 148,429 |
| 47,05 | 147,714 | 144,429 | 155 | 153,714 |
| 47 | 139,857 | 148,857 | 160,429 | 150,571 |
| 46,95 | 145,429 | 135,571 | 155,429 | 140,286 |
| 46,9 | 147,714 | 141 | 155,286 | 144,429 |
| 46,85 | 156,286 | 145,857 | 154,286 | 151 |
| 46,8 | 145,714 | 142,714 | 164,571 | 149,714 |
| 46,75 | 155,857 | 147,857 | 159,571 | 147,857 |
| 46,7 | 141,143 | 146 | 154,286 | 148 |
| 46,65 | 146 | 138,857 | 153,857 | 151,571 |
| 46,6 | 138,714 | 146 | 160,286 | 140,143 |
| 46,55 | 129,714 | 141 | 155 | 147,143 |
| 46,5 | 141,857 | 145,143 | 146,143 | 139,571 |
| 46,45 | 140,571 | 150,429 | 150,857 | 141,286 |
| 46,4 | 143,714 | 142,571 | 149,286 | 138,714 |
| 46,35 | 145,714 | 138,143 | 154,143 | 141,571 |
| 46,3 | 139,429 | 139,571 | 155,857 | 136,429 |
| 46,25 | 142,571 | 140,571 | 154,429 | 140,429 |
| 46,2 | 141,143 | 144,286 | 153,286 | 144,714 |
| 46,15 | 145,429 | 136,429 | 151,857 | 142,429 |
| 46,1 | 140,857 | 137,571 | 155,429 | 137,286 |
| 46,05 | 144,857 | 141,714 | 150,143 | 134 |
| 46 | 139,571 | 144,429 | 146,429 | 140,571 |

## Ti2p

| Binding energy [eV] | TNTs | Se-Low | Se-Medium | Se-High |
| --- | --- | --- | --- | --- |
| 470 | 805,333 | 806,889 | 944 | 1032,67 |
| 469,95 | 806,222 | 829,556 | 969,333 | 1022,67 |
| 469,9 | 811,556 | 821,111 | 957,778 | 1005,56 |
| 469,85 | 810 | 836,667 | 972,444 | 1013,11 |
| 469,8 | 804,444 | 804,667 | 938,444 | 1036,22 |
| 469,75 | 785,778 | 808,444 | 930,889 | 1016 |
| 469,7 | 803,111 | 797,333 | 940,889 | 1019,11 |
| 469,65 | 794,667 | 790,222 | 945,111 | 1002 |
| 469,6 | 809,778 | 799,556 | 953,333 | 1033,56 |
| 469,55 | 793,111 | 794,222 | 946 | 1027,11 |
| 469,5 | 794,667 | 786,222 | 937,111 | 1025,56 |
| 469,45 | 798,667 | 795,111 | 919,778 | 987,333 |
| 469,4 | 780,667 | 795,111 | 952,667 | 996,889 |
| 469,35 | 800,667 | 774,667 | 944,444 | 1008 |
| 469,3 | 801,556 | 776,667 | 946 | 1010,67 |
| 469,25 | 774,444 | 800 | 917,778 | 998,889 |
| 469,2 | 789,556 | 776,444 | 924,667 | 1028,44 |
| 469,15 | 766,222 | 784,889 | 910,667 | 1018,67 |
| 469,1 | 791,556 | 798,667 | 924 | 988 |
| 469,05 | 771,333 | 786,444 | 929,778 | 991,556 |
| 469 | 774,222 | 808,889 | 918,889 | 983,556 |
| 468,95 | 780,667 | 779,556 | 942 | 992,444 |
| 468,9 | 775,556 | 812,222 | 924,667 | 979,333 |
| 468,85 | 771,556 | 800 | 928 | 1000,89 |
| 468,8 | 787,333 | 786 | 936,444 | 992,667 |
| 468,75 | 761,333 | 778,444 | 919,333 | 1018,44 |
| 468,7 | 745,556 | 778,222 | 922,222 | 998,667 |
| 468,65 | 776,222 | 767,556 | 896,889 | 988,444 |
| 468,6 | 794,889 | 786,444 | 927,111 | 997,556 |
| 468,55 | 778,889 | 783,778 | 922,222 | 988,222 |
| 468,5 | 784,444 | 781,556 | 918 | 984,889 |
| 468,45 | 778 | 775,333 | 910,222 | 980,222 |
| 468,4 | 778,667 | 802,444 | 923,778 | 999,556 |
| 468,35 | 786,889 | 788,667 | 946,444 | 1002,89 |
| 468,3 | 792,667 | 789,333 | 952,444 | 1009,33 |
| 468,25 | 793,333 | 793,333 | 922,889 | 997,556 |
| 468,2 | 780,667 | 799,333 | 938,667 | 1014 |
| 468,15 | 772 | 796,667 | 937,556 | 1014,22 |
| 468,1 | 780,222 | 794,222 | 926 | 1006,89 |
| 468,05 | 773,111 | 813,111 | 914,889 | 1020,22 |
| 468 | 795,333 | 797,333 | 923,111 | 1034,89 |
| 467,95 | 798,667 | 814,667 | 941,333 | 1012,22 |
| 467,9 | 812,889 | 813,333 | 962,667 | 1003,56 |
| 467,85 | 811,111 | 812,667 | 950,889 | 1010,67 |
| 467,8 | 820,444 | 826 | 963,333 | 1014,44 |
| 467,75 | 839,556 | 821,778 | 953,556 | 1024,89 |
| 467,7 | 829,333 | 816,444 | 955,556 | 1038,89 |
| 467,65 | 834,222 | 832,889 | 980,222 | 1048,67 |
| 467,6 | 824 | 836,889 | 997,778 | 1056,89 |
| 467,55 | 838,667 | 845,778 | 994,889 | 1052,89 |
| 467,5 | 844,222 | 831,333 | 1018,22 | 1072 |
| 467,45 | 855,778 | 868,444 | 994,889 | 1058,89 |
| 467,4 | 865,333 | 851,333 | 1000,67 | 1080,89 |
| 467,35 | 893,333 | 854 | 1031,56 | 1074,67 |
| 467,3 | 859,778 | 864,444 | 1040,67 | 1064,67 |
| 467,25 | 898,222 | 899,111 | 1018,44 | 1096,22 |
| 467,2 | 896,889 | 905,333 | 1045,33 | 1100,89 |
| 467,15 | 912,889 | 919,556 | 1038,22 | 1116,89 |
| 467,1 | 906,444 | 932,889 | 1074,22 | 1114,67 |
| 467,05 | 945,333 | 924,667 | 1095,33 | 1148,67 |
| 467 | 978,889 | 954,444 | 1104,44 | 1163,11 |
| 466,95 | 971,778 | 988,667 | 1093,11 | 1168,89 |
| 466,9 | 977,778 | 1006,67 | 1130,44 | 1159,56 |
| 466,85 | 977,778 | 980,667 | 1142,22 | 1179,78 |
| 466,8 | 1001,56 | 990,667 | 1148 | 1217,78 |
| 466,75 | 1036,44 | 1013,11 | 1165,78 | 1234,89 |
| 466,7 | 1039,11 | 1052,67 | 1189,56 | 1226 |
| 466,65 | 1075,33 | 1054,44 | 1221,33 | 1245,33 |
| 466,6 | 1081,33 | 1050,89 | 1249,56 | 1280,22 |
| 466,55 | 1083,56 | 1078,44 | 1266,89 | 1294,22 |
| 466,5 | 1135,56 | 1147,11 | 1286 | 1324 |
| 466,45 | 1170,67 | 1152,22 | 1298 | 1361,11 |
| 466,4 | 1208,44 | 1168,22 | 1339,56 | 1382,67 |
| 466,35 | 1225,78 | 1209,11 | 1392,44 | 1415,33 |
| 466,3 | 1258,89 | 1220,67 | 1426,89 | 1432,89 |
| 466,25 | 1310,44 | 1280,22 | 1443,11 | 1459,33 |
| 466,2 | 1333,56 | 1325,78 | 1497,78 | 1512 |
| 466,15 | 1387,56 | 1361,78 | 1526,67 | 1530 |
| 466,1 | 1432,22 | 1380,67 | 1564 | 1603,11 |
| 466,05 | 1484,67 | 1448,44 | 1659,78 | 1614 |
| 466 | 1517,11 | 1482,22 | 1699,78 | 1678 |
| 465,95 | 1556,22 | 1536,67 | 1711,11 | 1733,56 |
| 465,9 | 1585,33 | 1551,78 | 1828,89 | 1756,89 |
| 465,85 | 1638,89 | 1608,89 | 1846,89 | 1778,89 |
| 465,8 | 1691,11 | 1656,67 | 1898 | 1845,78 |
| 465,75 | 1723,56 | 1684,89 | 1926,22 | 1858,22 |
| 465,7 | 1770 | 1750,22 | 1965,11 | 1924,22 |
| 465,65 | 1836,67 | 1779,78 | 2021,33 | 1964,89 |
| 465,6 | 1847,11 | 1794,22 | 2042 | 2003,33 |
| 465,55 | 1894 | 1868,44 | 2062,44 | 2044 |
| 465,5 | 1910,67 | 1880,44 | 2122,67 | 2046 |
| 465,45 | 1918,44 | 1902,22 | 2152,44 | 2097,56 |
| 465,4 | 1920,22 | 1927,56 | 2155,78 | 2136 |
| 465,35 | 1962,22 | 1919,56 | 2173,56 | 2136,44 |
| 465,3 | 2007,78 | 1968,67 | 2202,22 | 2156 |
| 465,25 | 2005,78 | 1944,44 | 2203,78 | 2132,89 |
| 465,2 | 1977,33 | 1971,78 | 2186,22 | 2150,44 |
| 465,15 | 1916,22 | 1965,78 | 2200,44 | 2153,11 |
| 465,1 | 1937,78 | 1944,89 | 2213,33 | 2171,33 |
| 465,05 | 1912,67 | 1933,78 | 2214,89 | 2174,67 |
| 465 | 1925,33 | 1895,11 | 2179,56 | 2138,89 |
| 464,95 | 1906,67 | 1881,56 | 2123,33 | 2101,56 |
| 464,9 | 1857,33 | 1875,11 | 2104,67 | 2094,22 |
| 464,85 | 1834,67 | 1869,33 | 2108,22 | 2096,67 |
| 464,8 | 1814,22 | 1823,11 | 2059,56 | 2038,22 |
| 464,75 | 1770,44 | 1836,44 | 2032,22 | 2009,56 |
| 464,7 | 1720,67 | 1788 | 1978,44 | 1968 |
| 464,65 | 1698,44 | 1698,22 | 1962 | 1930 |
| 464,6 | 1660,89 | 1656,67 | 1932,22 | 1896 |
| 464,55 | 1633,78 | 1612,22 | 1864,44 | 1853,78 |
| 464,5 | 1566,67 | 1581,11 | 1835,11 | 1781,11 |
| 464,45 | 1530 | 1517,33 | 1797,56 | 1763,11 |
| 464,4 | 1466,67 | 1484,44 | 1714,67 | 1732,22 |
| 464,35 | 1430,67 | 1463,11 | 1605,33 | 1679,78 |
| 464,3 | 1371,56 | 1400,67 | 1592,67 | 1645,78 |
| 464,25 | 1329,78 | 1352,22 | 1556 | 1596,44 |
| 464,2 | 1295,33 | 1327,11 | 1540,44 | 1558,89 |
| 464,15 | 1259,33 | 1294 | 1492,89 | 1525,56 |
| 464,1 | 1203,56 | 1258,44 | 1417,78 | 1479,56 |
| 464,05 | 1154,89 | 1229,33 | 1382,89 | 1426,22 |
| 464 | 1152,67 | 1164,22 | 1351,78 | 1384,44 |
| 463,95 | 1124 | 1127,78 | 1312,44 | 1333,11 |
| 463,9 | 1093,11 | 1095,56 | 1273,11 | 1335,11 |
| 463,85 | 1069,33 | 1069,78 | 1233,33 | 1282,22 |
| 463,8 | 1013,33 | 1044,22 | 1207,11 | 1282,67 |
| 463,75 | 993,556 | 1014,22 | 1168,22 | 1227,78 |
| 463,7 | 974,222 | 997,333 | 1128 | 1183,56 |
| 463,65 | 945,333 | 972,444 | 1115,56 | 1166,89 |
| 463,6 | 940,222 | 928,444 | 1099,78 | 1180,89 |
| 463,55 | 918,222 | 939,778 | 1102,44 | 1124,89 |
| 463,5 | 904,222 | 895,778 | 1075,78 | 1120 |
| 463,45 | 885,556 | 895,111 | 1040,89 | 1109,56 |
| 463,4 | 856,444 | 895,333 | 1015,33 | 1103,78 |
| 463,35 | 852,222 | 882 | 1012,22 | 1075,33 |
| 463,3 | 840 | 864,667 | 1020,89 | 1048,89 |
| 463,25 | 817,111 | 854,444 | 974,444 | 1066,89 |
| 463,2 | 824,222 | 856,444 | 961,778 | 1036 |
| 463,15 | 809,556 | 829,111 | 977,556 | 1038,89 |
| 463,1 | 791,556 | 832,889 | 940,667 | 1035,11 |
| 463,05 | 823,333 | 799,778 | 946 | 1038,67 |
| 463 | 797,111 | 794,889 | 945,111 | 996,889 |
| 462,95 | 807,333 | 784,222 | 919,778 | 1024 |
| 462,9 | 792,222 | 789,778 | 931,778 | 999,778 |
| 462,85 | 787,778 | 820 | 913,778 | 978,222 |
| 462,8 | 772,667 | 796,222 | 925,111 | 973,333 |
| 462,75 | 773,333 | 792,667 | 907,778 | 991,333 |
| 462,7 | 778,667 | 754,222 | 926,222 | 1000 |
| 462,65 | 776,222 | 752,889 | 903,333 | 968 |
| 462,6 | 758,889 | 770,444 | 920,222 | 962,444 |
| 462,55 | 786,667 | 784,667 | 924 | 964,667 |
| 462,5 | 761,111 | 794,444 | 935,111 | 990,667 |
| 462,45 | 766,667 | 778,889 | 911,111 | 974 |
| 462,4 | 783,556 | 784,889 | 888,667 | 964,222 |
| 462,35 | 792,222 | 772,444 | 902,667 | 973,111 |
| 462,3 | 772,444 | 773,111 | 915,556 | 992,222 |
| 462,25 | 778,222 | 765,333 | 911,556 | 989,333 |
| 462,2 | 788 | 790,444 | 889,111 | 995,778 |
| 462,15 | 774,444 | 788,444 | 913,556 | 1000,89 |
| 462,1 | 777,333 | 788,222 | 920,444 | 998 |
| 462,05 | 776,889 | 793,556 | 926,444 | 1008 |
| 462 | 794 | 806 | 937,111 | 992,889 |
| 461,95 | 809,556 | 810,889 | 907,556 | 1012,67 |
| 461,9 | 802,667 | 805,778 | 931,333 | 991,556 |
| 461,85 | 803,556 | 779,111 | 917,556 | 1002,22 |
| 461,8 | 831,556 | 803,778 | 939,556 | 1010 |
| 461,75 | 809,111 | 843,111 | 963,333 | 1022,22 |
| 461,7 | 830,889 | 836 | 978,889 | 1035,78 |
| 461,65 | 819,556 | 822,444 | 973,333 | 1020,44 |
| 461,6 | 828,444 | 829,333 | 964,667 | 1028 |
| 461,55 | 846,444 | 849,333 | 969,111 | 1033,33 |
| 461,5 | 850,444 | 851,111 | 985,333 | 1048,67 |
| 461,45 | 858 | 849,111 | 985,111 | 1052 |
| 461,4 | 845,111 | 860,222 | 1015,33 | 1064,67 |
| 461,35 | 844,667 | 884,667 | 1004 | 1061,78 |
| 461,3 | 883,111 | 874,667 | 1012,67 | 1052 |
| 461,25 | 888,444 | 866,444 | 1026,67 | 1062 |
| 461,2 | 890,667 | 894 | 1039,33 | 1107,56 |
| 461,15 | 900 | 895,778 | 1023,11 | 1105,56 |
| 461,1 | 910,222 | 885,556 | 1027,78 | 1080 |
| 461,05 | 904 | 895,556 | 1055,56 | 1104,89 |
| 461 | 927,333 | 880,889 | 1040 | 1105,56 |
| 460,95 | 949,111 | 909,556 | 1047,33 | 1126 |
| 460,9 | 972 | 952,222 | 1070 | 1128,44 |
| 460,85 | 972,222 | 969,556 | 1090,44 | 1142 |
| 460,8 | 978,889 | 972,889 | 1100,89 | 1166,22 |
| 460,75 | 1016,89 | 990,667 | 1147,78 | 1173,78 |
| 460,7 | 1055,11 | 1018,22 | 1164,44 | 1205,11 |
| 460,65 | 1100 | 1058 | 1223,78 | 1224,89 |
| 460,6 | 1128,22 | 1094,22 | 1255,33 | 1267,11 |
| 460,55 | 1184 | 1134,44 | 1283,11 | 1340,44 |
| 460,5 | 1274,22 | 1206,89 | 1348 | 1382,89 |
| 460,45 | 1334,67 | 1248,67 | 1427,78 | 1456,22 |
| 460,4 | 1415,56 | 1344,22 | 1494,22 | 1522,44 |
| 460,35 | 1516,89 | 1423,56 | 1615,33 | 1597,78 |
| 460,3 | 1642,22 | 1546,22 | 1741,11 | 1672 |
| 460,25 | 1787,78 | 1671,56 | 1879,78 | 1802,22 |
| 460,2 | 1944,89 | 1829,11 | 2058,44 | 1968,67 |
| 460,15 | 2150,89 | 1997,33 | 2240,89 | 2106,44 |
| 460,1 | 2354 | 2174,67 | 2435,33 | 2295,78 |
| 460,05 | 2561,56 | 2415,78 | 2646 | 2514,22 |
| 460 | 2828,22 | 2625,78 | 2930,22 | 2727,78 |
| 459,95 | 3089,78 | 2893,56 | 3186 | 2985,33 |
| 459,9 | 3354,67 | 3190,67 | 3464,67 | 3197,33 |
| 459,85 | 3610,89 | 3413,11 | 3788,22 | 3534,89 |
| 459,8 | 3876,22 | 3741,33 | 4112,89 | 3756,44 |
| 459,75 | 4164 | 3982 | 4491,78 | 4094,67 |
| 459,7 | 4478,89 | 4310,67 | 4724 | 4339,33 |
| 459,65 | 4704 | 4528,44 | 5054,44 | 4644 |
| 459,6 | 4901,33 | 4754 | 5282,89 | 4865,11 |
| 459,55 | 5050,89 | 4917,56 | 5452,89 | 5037,78 |
| 459,5 | 5163,78 | 5115,33 | 5614,44 | 5224,44 |
| 459,45 | 5241,78 | 5164,89 | 5688,89 | 5330 |
| 459,4 | 5221,11 | 5159,33 | 5721,33 | 5371,56 |
| 459,35 | 5128,44 | 5128 | 5658,44 | 5333,78 |
| 459,3 | 4974,67 | 4965,78 | 5593,56 | 5278,89 |
| 459,25 | 4788,67 | 4828,89 | 5360,22 | 5128,89 |
| 459,2 | 4540,44 | 4570,22 | 5148,22 | 4873,78 |
| 459,15 | 4175,78 | 4294,89 | 4846,22 | 4670 |
| 459,1 | 3893,78 | 3979,78 | 4491,33 | 4414,67 |
| 459,05 | 3532,44 | 3724,22 | 4120,22 | 4081,56 |
| 459 | 3195,33 | 3354,44 | 3760,89 | 3746,89 |
| 458,95 | 2896,89 | 3014,89 | 3429,33 | 3425,33 |
| 458,9 | 2540,44 | 2690,44 | 3095,11 | 3086,67 |
| 458,85 | 2228,89 | 2367,56 | 2749,56 | 2760,44 |
| 458,8 | 1959,78 | 2093,78 | 2433,78 | 2482,89 |
| 458,75 | 1760,89 | 1886 | 2164,44 | 2214,67 |
| 458,7 | 1508,89 | 1682,67 | 1928,89 | 1978,22 |
| 458,65 | 1323,33 | 1478,44 | 1705,11 | 1772,89 |
| 458,6 | 1200,67 | 1313,11 | 1479,11 | 1596,44 |
| 458,55 | 1064,67 | 1168 | 1360,44 | 1462,89 |
| 458,5 | 965,333 | 1055,56 | 1229,78 | 1334,89 |
| 458,45 | 872,444 | 966 | 1151,56 | 1229,78 |
| 458,4 | 784 | 880,222 | 1044,67 | 1112,22 |
| 458,35 | 752,444 | 813,111 | 944,667 | 1052,44 |
| 458,3 | 714,667 | 758,444 | 882,444 | 994,889 |
| 458,25 | 662 | 705,333 | 843,111 | 923,333 |
| 458,2 | 614,667 | 657,778 | 784,667 | 899,333 |
| 458,15 | 606,222 | 624,444 | 742,667 | 876,222 |
| 458,1 | 560,667 | 606 | 726,889 | 835,556 |
| 458,05 | 549,556 | 582,667 | 702,222 | 801,333 |
| 458 | 529,778 | 541,111 | 672 | 767,556 |
| 457,95 | 520,222 | 540,222 | 666 | 756,222 |
| 457,9 | 496,889 | 532,444 | 632 | 747,556 |
| 457,85 | 489,111 | 506,222 | 612,222 | 732,889 |
| 457,8 | 494,667 | 509,333 | 613,556 | 709,778 |
| 457,75 | 483,556 | 486,667 | 606,444 | 696,222 |
| 457,7 | 467,778 | 479,333 | 593,333 | 682,667 |
| 457,65 | 464,222 | 465,111 | 592,444 | 669,111 |
| 457,6 | 446,667 | 460,667 | 557,556 | 661,556 |
| 457,55 | 437,333 | 453,111 | 563,333 | 669,333 |
| 457,5 | 435,778 | 444,222 | 550 | 664 |
| 457,45 | 416,222 | 440,667 | 545,111 | 654,222 |
| 457,4 | 410 | 436,667 | 533,111 | 658,667 |
| 457,35 | 411,556 | 420,889 | 519,111 | 651,778 |
| 457,3 | 396,444 | 419,333 | 517,111 | 624,222 |
| 457,25 | 375,333 | 410,444 | 487,556 | 618,889 |
| 457,2 | 372,444 | 388,222 | 476,889 | 592,222 |
| 457,15 | 375,556 | 385,556 | 485,111 | 598,889 |
| 457,1 | 364,667 | 386 | 476,444 | 588 |
| 457,05 | 343,111 | 366 | 455,556 | 562,667 |
| 457 | 343,333 | 362,222 | 452,444 | 563,111 |
| 456,95 | 319,111 | 358,444 | 440,444 | 572,667 |
| 456,9 | 319,778 | 350 | 437,556 | 552 |
| 456,85 | 311,778 | 333,333 | 423,556 | 547,111 |
| 456,8 | 301,778 | 323,333 | 403,556 | 542,222 |
| 456,75 | 297,556 | 325,111 | 402 | 530,222 |
| 456,7 | 294,667 | 310,667 | 386,889 | 523,556 |
| 456,65 | 284,667 | 303,333 | 381,556 | 530,444 |
| 456,6 | 283,778 | 305,333 | 376 | 516,444 |
| 456,55 | 282,444 | 281,778 | 372 | 508,444 |
| 456,5 | 277,111 | 286,222 | 365,556 | 497,556 |
| 456,45 | 263,778 | 293,111 | 376,222 | 510,444 |
| 456,4 | 263,111 | 286 | 357,778 | 485,556 |
| 456,35 | 254,444 | 274,222 | 376,444 | 521,111 |
| 456,3 | 240,222 | 270 | 353,556 | 501,556 |
| 456,25 | 236 | 273,111 | 342 | 488,444 |
| 456,2 | 250,444 | 278 | 340 | 496,444 |
| 456,15 | 241,111 | 271,778 | 344,444 | 483,111 |
| 456,1 | 237,333 | 263,778 | 350,667 | 482 |
| 456,05 | 236,222 | 270 | 319,111 | 492,222 |
| 456 | 231,556 | 260,667 | 324,222 | 462,444 |
| 455,95 | 231,333 | 256,667 | 336,667 | 466,667 |
| 455,9 | 218,667 | 250 | 331,556 | 478 |
| 455,85 | 224,444 | 241,778 | 338,444 | 483,333 |
| 455,8 | 224,667 | 260,222 | 326 | 480,667 |
| 455,75 | 228,444 | 255,111 | 326 | 485,333 |
| 455,7 | 225,111 | 243,778 | 326,667 | 479,556 |
| 455,65 | 220,667 | 249,333 | 327,778 | 461,333 |
| 455,6 | 230,889 | 256 | 327,333 | 468,889 |
| 455,55 | 216 | 258,667 | 334,889 | 483,111 |
| 455,5 | 228 | 248,667 | 315,556 | 464,889 |
| 455,45 | 228,667 | 254 | 318,444 | 466,222 |
| 455,4 | 213,111 | 249,333 | 320,889 | 468,222 |
| 455,35 | 223,556 | 239,556 | 327,333 | 466,222 |
| 455,3 | 230,444 | 239,556 | 335,111 | 464,444 |
| 455,25 | 218 | 246,667 | 318 | 462,222 |
| 455,2 | 217,778 | 249,111 | 317,778 | 472,889 |
| 455,15 | 207,556 | 244,444 | 316,889 | 466,667 |
| 455,1 | 213,111 | 240,889 | 316,444 | 452,667 |
| 455,05 | 208,667 | 245,111 | 312,222 | 464 |
| 455 | 210,444 | 250,889 | 322,889 | 474,444 |
| 454,95 | 213,556 | 247,111 | 322,444 | 482,667 |
| 454,9 | 215,111 | 246 | 323,111 | 456,667 |
| 454,85 | 214,444 | 259,556 | 317,333 | 462,222 |
| 454,8 | 217,556 | 244,889 | 316,444 | 460 |
| 454,75 | 218,444 | 235,778 | 310,889 | 462 |
| 454,7 | 212 | 238,444 | 318 | 466,222 |
| 454,65 | 207,111 | 241,556 | 319,556 | 446 |
| 454,6 | 213,778 | 234,444 | 316,222 | 448,667 |
| 454,55 | 217,333 | 242,444 | 312,444 | 474,889 |
| 454,5 | 214,889 | 232,444 | 311,111 | 447,556 |
| 454,45 | 212 | 241,778 | 302 | 437,111 |
| 454,4 | 212,667 | 234 | 318,444 | 458,444 |
| 454,35 | 202,889 | 230,222 | 316 | 446,889 |
| 454,3 | 212,444 | 226,444 | 312 | 456 |
| 454,25 | 213,333 | 243,556 | 310,889 | 458 |
| 454,2 | 200,222 | 232 | 302,222 | 457,778 |
| 454,15 | 210 | 227,333 | 303,778 | 455,111 |
| 454,1 | 210,222 | 234,444 | 317,778 | 458,889 |
| 454,05 | 206 | 232 | 315,556 | 455,333 |
| 454 | 210,667 | 238,667 | 315,778 | 443,556 |
| 453,95 | 219,556 | 243,111 | 303,778 | 456 |
| 453,9 | 214,889 | 240 | 298,889 | 460,667 |
| 453,85 | 213,333 | 228 | 298 | 456 |
| 453,8 | 209,556 | 233,333 | 295,556 | 462 |
| 453,75 | 214,889 | 233,556 | 294,222 | 470,667 |
| 453,7 | 208,889 | 246 | 306,222 | 468 |
| 453,65 | 209,111 | 245,556 | 301,778 | 465,333 |
| 453,6 | 210,889 | 237,111 | 293,778 | 463,333 |
| 453,55 | 199,333 | 239,778 | 302,222 | 461,333 |
| 453,5 | 204,444 | 231,333 | 310,889 | 430 |
| 453,45 | 212,222 | 232 | 298,889 | 451,556 |
| 453,4 | 214,444 | 226,667 | 301,556 | 452,444 |
| 453,35 | 206,222 | 232,667 | 307,778 | 451,556 |
| 453,3 | 192 | 233,111 | 327,556 | 472 |
| 453,25 | 203,556 | 240,444 | 320,222 | 460,444 |
| 453,2 | 202,667 | 223,111 | 313,111 | 449,556 |
| 453,15 | 208,444 | 220,889 | 309,556 | 446,667 |
| 453,1 | 205,778 | 227,333 | 306 | 452,889 |
| 453,05 | 214 | 233,556 | 310,444 | 447,111 |
| 453 | 209,333 | 235,556 | 309,778 | 458,667 |

# Dataset- ICP MS – Se release [ppb]

| Day | Se-Low | Se-Medium | Se-High |
| --- | --- | --- | --- |
| 2 | 15,30534 ± 2,12571 | 18,14874 ± 2,05707 | 36,66044 ± 1,88345 |
| 5 | 20,26605 ± 0,58946 | 22,92797 ± 1,19598 | 43,49619 ± 1,01673 |
| 9 | 23,10927 ± 0,7595 | 29,58242 ± 0,70941 | 47,36803 ± 0,9175 |
| 12 | 25,16597 ± 0,83953 | 31,45764 ± 1,30797 | 49,06176 ± 1,10297 |
| 15 | 26,79927 ± 0,87129 | 33,03033 ± 0,91729 | 51,30012 ± 0,50605 |

# Dataset – XTT 450 nm– Viability

## MG-63

|  | 24H | | | 48H | | | 6 days | | |
| --- | --- | --- | --- | --- | --- | --- | --- | --- | --- |
| TNTs | 0,082 | 0,079 | 0,093 | 0,095 | 0,099 | 0,098 | 0,208 | 0,171 | 0,191 |
| Se-Low | 0,054 | 0,059 | 0,043 | 0,054 | 0,059 | 0,034 | 0,087 | 0,07 | 0,04 |
| Se-Medium | 0,024 | 0,038 | 0,028 | 0,01 | 0,019 | 0,014 | 0,018 | 0,028 | 0,055 |
| Se-High | 0,004 | 0 | 0,012 | 0,005 | 0,006 | 0,004 | 0 | 0,014 | 0,014 |

t-test (p<0.05 considered important) 24H

|  | TNTs | Se-Low | Se-Medium | Se-High |
| --- | --- | --- | --- | --- |
| TNTs | X | X | X | X |
| Se-Low | 0,0068 | X | X | X |
| Se-Medium | 0,0008 | 0,0250 | X | X |
| Se-High | 0,0001 | 0,0013 | 0,0107 | X |

t-test (p<0.05 considered important) 48H

|  | TNTs | Se-Low | Se-Medium | Se-High |
| --- | --- | --- | --- | --- |
| TNTs | X | X | X | X |
| Se-Low | 0,0033 | X | X | X |
| Se-Medium | 8E-06 | 0,0127 | X | X |
| Se-High | 2,61E-07 | 0,0045 | 0,0248 | X |

t-test (p<0.05 considered important) 6 days

|  | TNTs | Se-Low | Se-Medium | Se-High |
| --- | --- | --- | --- | --- |
| TNTs | X | X | X | X |
| Se-Low | 0,0020 | X | X | X |
| Se-Medium | 0,0005 | 0,1437 | X | X |
| Se-High | 0,0001 | 0,0178 | 0,1124 | X |

## NIH/3T3

|  | 24H | | | 48H | | | 6 days | | |
| --- | --- | --- | --- | --- | --- | --- | --- | --- | --- |
| TNTs | 0,081 | 0,069 | 0,081 | 0,092 | 0,082 | 0,087 | 0,249 | 0,234 | 0,268 |
| Se-Low | 0,074 | 0,081 | 0,067 | 0,078 | 0,083 | 0,074 | 0,195 | 0,212 | 0,244 |
| Se-Medium | 0,079 | 0,056 | 0,081 | 0,082 | 0,067 | 0,067 | 0,151 | 0,134 | 0,177 |
| Se-High | 0,011 | 0,013 | 0,024 | 0,004 | 0,001 | 0,009 | 0,014 | 0,036 | 0,015 |

t-test (p<0.05 considered important) 24H

|  | TNTs | Se-Low | Se-Medium | Se-High |
| --- | --- | --- | --- | --- |
| TNTs | X | X | X | X |
| Se-Low | 0,6257 | X | X | X |
| Se-Medium | 0,6067 | 0,8347 | X | X |
| Se-High | 0,0004 | 0,0005 | 0,0034 | X |

t-test (p<0.05 considered important) 48H

|  | TNTs | Se-Low | Se-Medium | Se-High |
| --- | --- | --- | --- | --- |
| TNTs | X | X | X | X |
| Se-Low | 0,8966 | X | X | X |
| Se-Medium | 0,0600 | 0,3241 | X | X |
| Se-High | 2,45E-05 | 3E-05 | 0,0003 | X |

t-test (p<0.05 considered important) 6 days

|  | TNTs | Se-Low | Se-Medium | Se-High |
| --- | --- | --- | --- | --- |
| TNTs | X | X | X | X |
| Se-Low | 0,1281 | X | X | X |
| Se-Medium | 0,0038 | 0,0297 | X | X |
| Se-High | 4,73E-05 | 0,0003 | 0,0007 | X |

# Dataset – Bacteria colony count [ CFU ml^-1^]

| TNTs | Se-Low | Se-Medium | Se-High |
| --- | --- | --- | --- |
| 673000 | 286000 | 99000 | 66000 |
| 711000 | 312000 | 118500 | 76500 |
| 697500 | 303500 | 111500 | 71000 |

|  | N | Mean | Standard deviation |
| --- | --- | --- | --- |
| TNTs | 3 | 693833,3333 | 19263,52339 |
| Se-Low | 3 | 300500 | 13257,07358 |
| Se-Medium | 3 | 109666,7 | 9878,428 |
| Se-High | 3 | 71166,67 | 5251,984 |

t-test (p<0.05 considered important)

|  | TNTs | Se-Low | Se-Medium | Se-High |
| --- | --- | --- | --- | --- |
| TNTs | X | X | X | X |
| Se-Low | 8,2637E-06 | X | X | X |
| Se-Medium | 1,2536E-06 | 3,6938E-05 | X | X |
| Se-High | 7,0326E-07 | 9,8795E-06 | 0,00397 | X |

# Dataset – Contact angle

## TNTs


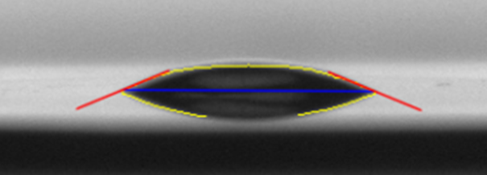


## Se-Low


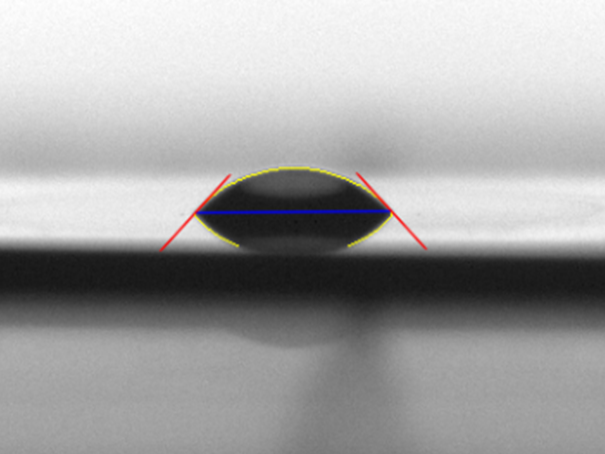


## Se-Medium


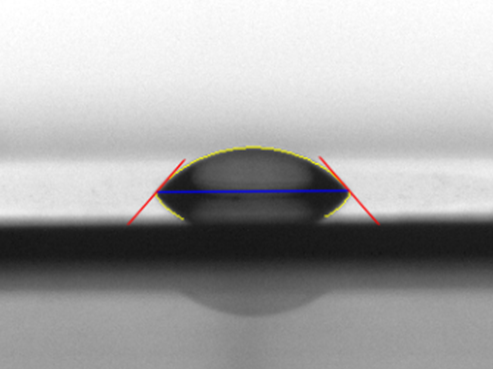


## Se-High


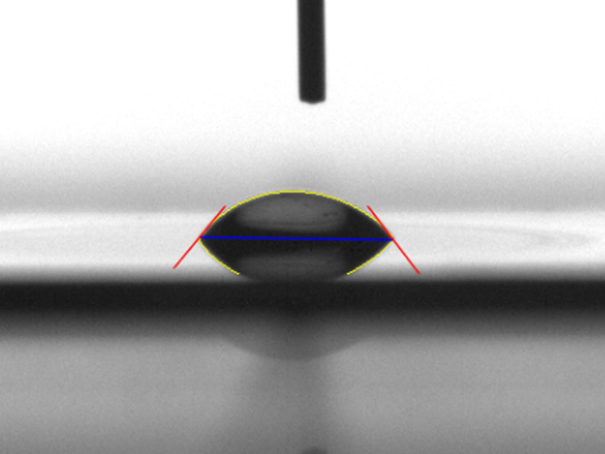


# Dataset – Bacterial colonies – Agar plates

## TNTS


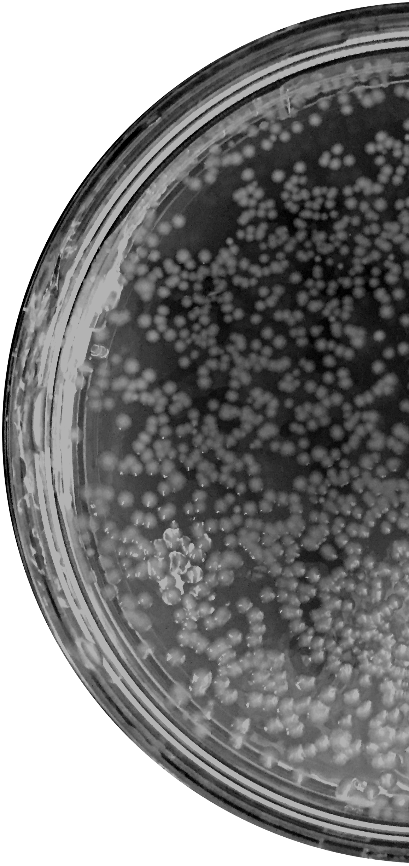


## Se-Low


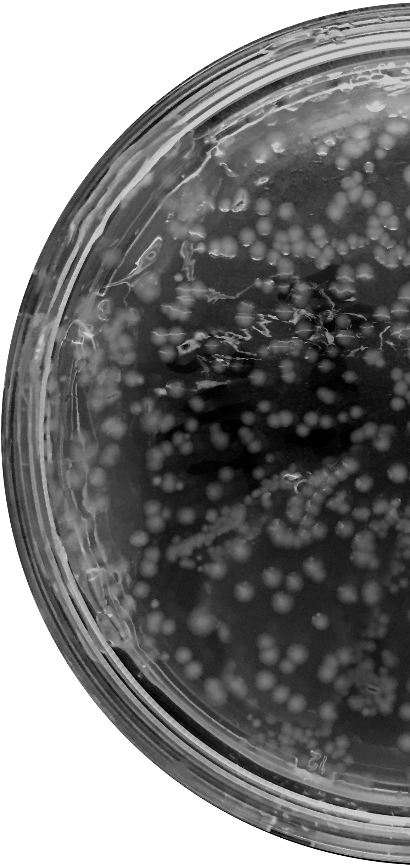


## Se-Medium


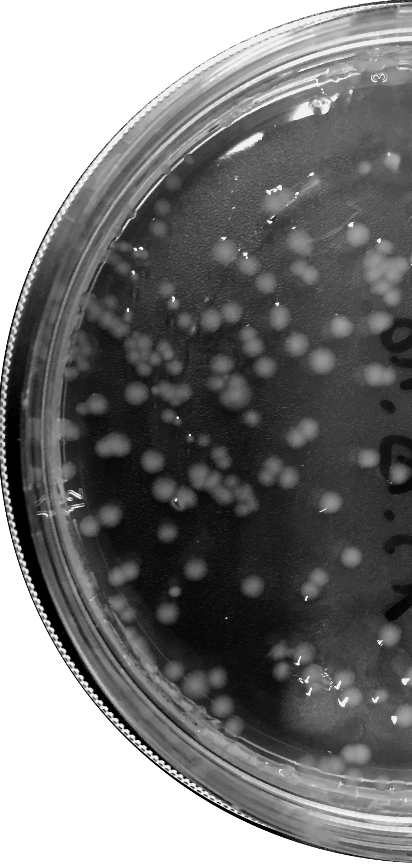


## Se-High


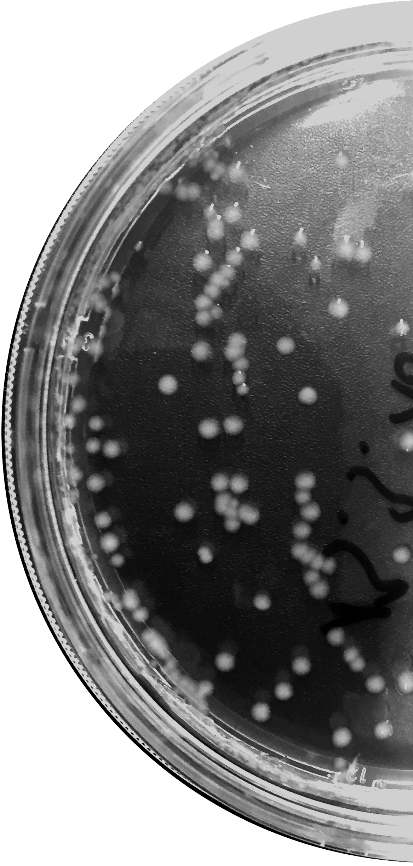

Supplement: S1 File — File contains raw data from which the graphs were drawn. (DOCX) [file pone.0214066.s001.docx]
